# Supplementary material for: Biodesulfurization Induces Reprogramming of Sulfur Metabolism in Rhodococcus qingshengii IGTS8: Proteomics and Untargeted Metabolomics
Source: Microbiol Spectr. 2021 Sep 1;9(2):e00692-21. doi: 10.1128/Spectrum.00692-21 (PMC8557817; doi:10.1128/Spectrum.00692-21)
Supplement: SUPPLEMENTAL FILE 1 — Supplemental material. Download SPECTRUM00692-21_Supp_1_seq12.pdf, PDF file, 1.5 MB. [file spectrum00692-21_supp_1_seq12.pdf]

This supplemental file includes:

Supplemental Results and Discussion

Supplemental Figures S1-S8

Supplemental Tables S1 and S4

Sulfur Sparing Analysis (Protein sequences)

Tables S2 and S3 are submitted separately

Results

Growth of *R. qingshengii* IGTS8 on dibenzothiophene and inorganic sulfate

We performed comparative systems biology studies; namely, proteomics and untargeted metabolomics, on the biodesulfurization reference strain *R. qingshengii* IGTS8 to gain insight into adaptations of assimilatory sulfur metabolism under biodesulfurization conditions. Strain IGTS8 grew in chemically defined medium containing either inorganic sulfate (sulfate-sufficient culture) or dibenzothiophene (sulfate-deficient, biodesulfurizing culture) as the sole sulfur source and exhibited logarithmic growth after a lag phase of varying duration (~20 h and 30 h for the sulfate and dibenzothiophene culture, respectively) (Fig. S2). The growth parameters differed depending on the sulfur source. While a specific growth rate (μ) of 0.18 ± 0.03 h^{-1} (generation time 5.6 ± 0.1 h) was observed on sulfate, growth on dibenzothiophene resulted in a lower specific growth rate of 0.09 ± 0.01 h^{-1} and, accordingly, longer generation time of 11 ± 1.4 h.

A global look at the sulfur proteome and metabolome

Based on genomic analysis, 101 proteins were predicted to be related to assimilatory sulfur metabolism in the IGTS8 strain (Table S2). As shown in Table S1, we added other sulfur metabolism-associated proteins to cover the biosynthesis of low-molecular weight thiols, sulfur-containing cofactors and vitamins, siroheme, and phosphoserine, resulting in a total of 125 sulfur metabolism-related proteins. Of these, 111 (90%) were detected in the *R. qingshengii* IGTS8 proteome and 52 exhibited significantly different abundance at least during one growth phase. Sixteen proteins were uniquely detected and 47 were significantly more abundant in the dibenzothiophene culture at least during one growth phase. All key pathways related to assimilatory sulfur metabolism were represented in the sulfur proteome including uptake of the

sulfur sources, sulfur acquisition, assimilatory sulfate reduction, biosynthesis of key sulfur-containing metabolites such as S-adenosylmethionine, coenzyme A (CoA), biotin, thiamin, molybdenum cofactor (MoCo), mycothiol and ergothioneine (low-molecular weight thiols), biogenesis of Fe-S clusters, transcriptional regulation, oxidative stress (redox homeostasis) and sulfur relay pathways. Components of uptake systems and sulfur acquisition proteins, including those of the 4S biodesulfurization pathway, which constitute a major part of the sulfate starvation-induced proteome, exhibited the strongest change in protein abundance. In addition, proteins of oxidative stress response such as alkylhydroperoxide reductase and thiol peroxidase were more abundant under biodesulfurization conditions.

Dibenzothiophene stimulates the production of sulfate starvation-induced proteins

In addition to the key sulfur metabolism enzymes, the sulfur proteome revealed a number of differentially expressed proteins, which are known to be induced under sulfate-limiting/starvation conditions and collectively known as sulfate starvation-induced (SSI) proteins. The majority of these proteins are those needed for the uptake of, and sulfur acquisition from, low-preference (alternative) sulfur sources, particularly ABC-type transporters, sulfatases and oxygenases including the 4S pathway enzymes (Dsz enzymes) (Table S1).

Subunits of an alkanesulfonate monooxygenase SsuD and SsuA (IGTS8_peg4802 and IGTS8_peg4804) were uniquely present in the dibenzothiophene culture during different growth phases and the encoding genes are co-expressed with other related genes and alkanesulfonate transporters. This gene cluster encodes proteins for transport and metabolism of aliphatic sulfonate esters (the Ssu operon, sulfonate sulfur utilization), which is usually induced under sulfate-limiting conditions. The IclR family transcriptional regulator of this operon is divergently encoded upstream.

Another SSI protein, α-ketoglutarate-dependent taurine dioxygenase (TauD, IGTS8_peg5939), was uniquely detected in the dibenzothiophene culture most of the time. By looking into the genomic context of the IGTS8_peg5939 gene, we found a close by gene encoding a choline sulfatase (AtsA, IGTS8_peg5938), which was similarly uniquely present in the dibenzothiophene culture during the mid-log, late-log and stationary phases (Table S1). The protein sequence of IGTS8_peg5938 exhibits a couple of conserved peptide signatures specific to choline sulfatases namely, GKWHL and CSPSR (1). The two genes constitute an operon regulated

by an ROK family transcriptional regulator (IGTS8_peg5940) that is encoded on the reverse direction and was very strongly upregulated in the dibenzothiophene-grown cells particularly during the stationary phase.

Many flavin-utilizing monooxygenases were strongly upregulated in the dibenzothiophene culture (Table S1). Two of them (IGTS8_peg5732, IGTS8_peg3535) belong to the bacterial luciferase family and their protein sequences are identical to nitrilotriacetate monooxygenase and dimethylsulfide monooxygenase from different bacteria. IGTS8_peg5732 was uniquely detected in the dibenzothiophene culture during the early-log and late-log phases. Furthermore, dibenzothiophene triggered the expression of a homolog of dimethylsulfone monooxygenase SfnG (IGTS8_peg4243), a key enzyme of dimethylsulfide desulfurization.

Several oxidative stress proteins such as alkylhydroperoxide reductase (IGTS8_peg1416, (IGTS8_peg1417) and thiol peroxidase (IGTS8_peg2514) were upregulated under biodesulfurization conditions, whereas a protein-disulfide isomerase (DsbA) was downregulated in the dibenzothiophene culture (Table S1). Furthermore, the proteome data revealed many thiosulfate sulfurtransferase (rhodaneses) homologs. However, these were either downregulated in the dibenzothiophene culture or having no significant difference in the protein level between the dibenzothiophene and sulfate cultures (Table S1).

Potential 2-hydroxybiphenyl efflux proteins

The proteome of the dibenzothiophene culture of IGTS8 contained some proteins that could be potential efflux systems for the biodesulfurization end product 2-hydroxybiphenyl (Table S1). The level of KefA (IGTS8_peg999), a mechanosensitive channel, was significantly higher in the dibenzothiophene cultures from the mid-log and late-log phases (Log_2 fold change = 6.0 and 4.5, respectively). In addition, the biodesulfurizing culture produced up to 14-fold (Log_2 fold change = 2.4-3.8) higher level of the ATPase component (DrrA_5, IGTS8_peg1647) of an ABC-type multidrug transport system than the sulfate culture. To date, it is not known how 2-hydroxybiphenyl is excreted from the biodesulfurizing cells. However, the MexAB-OprM efflux system was shown to play a role in the excretion of 2-hydroxybiphenyl in the 2-hydroxybiphenyl-degrading *Pseudomonas azelaica* HBP1 (2).

Cysteine biosynthesis

Enzymes of phosphoserine biosynthesis (an alternative cysteine precursor in *Mycobacterium tuberculosis*) were detected in the proteome with different abundancies between the dibenzothiophene and sulfate cultures, but changes were mostly in the statistically non-significant range (Table S1). Phosphoserine was not detected in the metabolome of any of the cultures. Instead, a metabolite annotated as *O*-phosphohomoserine, a precursor of methionine in plants, was more abundant in the dibenzothiophene culture, particularly during the mid- and late-log phases (Table S3). This finding is in line with the detection of homoserine kinase ThrB (IGTS8_peg2566) that was also more abundant in the dibenzothiophene culture (Table S1). Downstream of *thrB* and in the same direction, the two genes IGTS8_peg2567 and IGTS8_peg2568 encode threonine synthase (ThrC) and homoserine dehydrogenase (Hom) which were also detected in the proteome (Table S1). The exclusive detection of the putative IGTS8_peg5732 monooxygenase in the dibenzothiophene culture questions its potential role under biodesulfurization conditions. BLAST search using Uniprot revealed that this protein is 48.6% identical (E-value: 2.6e-148) to *N*-acetyl-S-(2-succino)-L-cysteine monooxygenase from *Bacillus subtilis* strain 168 (UniProt KB-P54950, SCMK-BACSU). This enzyme catalyzes the oxygenation of the 2-position of the succinyl moiety of *N*-acetyl-S-(2-succino)-L-cysteine, causing a spontaneous elimination reaction of the resulting hemithioketal that generates oxaloacetate and *N*-acetylcysteine. This reaction is part of the S-(2-succino)-L-cysteine (2SC) degradation pathway that enables *B. subtilis* to grow on it as a sole sulfur source via cysteine, which allows the utilization of (2SC) as a sulfur source (3). Metabolism of 2SC produces cysteine, thus making this enzyme a part of cysteine biosynthesis (3).

Mycothiol-dependent detoxification enzymes

The IGTS8 proteome revealed enzymes that catalyze mycothiol-dependent reactions of detoxification. For instance, the expression level of a protein annotated as formaldehyde dehydrogenase NAD/mycothiol-dependent/S-nitrosomycothiol reductase MscR (IGTS8_peg4197) was slightly higher in the dibenzothiophene culture and increased towards the stationary phase, but at no significant difference compared to the inorganic sulfate culture (Table S1). This protein is 99.4% identical (E-value: 0.0) to S-(hydroxymethyl)mycothiol dehydrogenase from *Rhodococcus* sp. strain AD45. Directly upstream of IGTS8_peg4197 and in the same

direction a putative hydrolase (IGTS8_peg4196) was expressed and was mostly significantly more abundant in the sulfate culture, accumulating toward the stationary phase. This protein is 90% identical (E-value: 4.5e-139) to a MBL (metallo-β-lactamase)-fold metallohydrolase from the lactamase_B protein family. In contrast, in the presence of dibenzothiophene, the abundance of Mtr (a mycothione reductase) and Mca (a mycothiol S-conjugate amidase) appeared to increase with the incubation time while remaining not significantly different as compared to the sulfate culture.

Cofactor metabolism

Some of the key enzymes and metabolites involved in the metabolism of sulfur-containing vitamins and cofactors exhibited differential abundance based on sulfate availability. For instance, enzymes of pantothenate and CoA biosynthesis such as ketopantoate reductase (PanG) and pantothenate kinase (CoaA), were significantly upregulated in the biodesulfurizing culture during the stationary (Log_2 fold change = 2.4) and early-log (Log_2 fold change = 2.2) phases, respectively (Fig. S3C, Tables 1 and S1). We also detected phosphopantetheine adenylyltransferase (CoaD), dephospho-CoA kinase (CoaE), pantoate-β-alanine ligase (PanC) and bifunctional phosphopantothenoylcysteine decarboxylase/phosphopantothenoylcysteine synthetase (CoaBC) (Fig. S3C, Table S1). The level of these enzymes was slightly higher in the dibenzothiophene cultures and generally increased toward the stationary phase, albeit at no significant difference compared to the inorganic sulfate cultures. Among the CoA and pantothenate biosynthetic metabolites, we detected two intermediates, namely; pantothenate and dephospho-CoA. The level of pantothenate was not significantly different between the sulfate and dibenzothiophene cultures. However, dephospho-CoA was significantly more abundant in the sulfate culture during the mid-log and late-log phases (Table S3). Coenzyme A was enriched in the biodesulfurizing culture during the early-log phase (Log_2 fold change = 1.8) and then there was a downshift in its level (Fig. S4).

As shown in Figure S5 and Table S1, enzymes of molybdenum cofactor (MoCo) biosynthesis were detected in the IGTS8 proteome (4). Biodesulfurization stimulated the synthesis of MoaB (Log_2 fold change = 2.1-2.9), a key enzyme of molybdenum cofactor biosynthesis. For two other enzymes, MoaCB and two copies of MoeA, although the Log_2 fold change (0.8-1.6) appeared higher in the biodesulfurizing culture, it remained not significantly different from that of

the sulfate culture. In addition, cyclic pyranopterin monophosphate (precursor Z) was ~ two to fourfold more abundant in the sulfate culture (Fig. S5, Table S3). Precursor Z and the enzyme MoaCB constitute a part of the sulfur relay system. Furthermore, the dibenzothiophene culture had a significantly higher content of 2-iminoacetate, a key metabolite of thiamin metabolism, which was even uniquely present in the dibenzothiophene culture during the mid-log and stationary phases (Figs. S4 and S6, Table S3).

Several enzymes of thiamin metabolism were detected with an overall pattern of increasing from the early-log phase toward the stationary phase (Fig. S6, Table S1). Two other enzymes were not detected in the proteome, i.e. ThiE (thiaminphosphate pyrophosphorylase) and ThiS (sulfur carrier protein), albeit homologs of the encoding genes are present in the IGTS8 genome. Enzymes that produce 2-iminoacetate could not be detected in the proteome. One of them, the S-adenosylmethionine-dependent 2-iminoacetate synthase (ThiH) that produces 2-iminoacetate from tyrosine in *Escherichia coli* has no homolog in the IGTS8 strain, while the gene encoding the other enzyme that produces 2-iminoacetate in *Bacillus subtilis*, glycine oxidase (ThiO) is present in the IGTS8 genome. In contrast to 2-iminoacetate, thiamin monophosphate was mostly more abundant in the sulfate culture. Thiamin monophosphate is produced by thiamin-phosphate pyrophosphorylase (thiamin phosphate synthase, ThiE) and is then phosphorylated by thiamin monophosphate kinase (ThiL) leading to the final product thiamin diphosphate (TPP) (Table S1).

Biotin was detected in the metabolome of both the dibenzothiophene and sulfate cultures, albeit with a higher content in the sulfate-grown cells (Log_2 fold change = -1.07 to -2.04) which peaked during the stationary phase (Fig. S4, Table S3). Some enzymes of biotin biosynthesis were identified in the proteome and their levels were not significantly different between the dibenzothiophene and sulfate cultures (Fig. S7, Table S1). No metabolites of biotin biosynthesis were detected in the metabolome (5). Only the spontaneous oxidation product of biotin, biotin sulfoxide, was identified. Similar to biotin, biotin sulfoxide was 1- to 2-fold more abundant in the sulfate culture, however with a temporal pattern the opposite of that observed for biotin (Table S3). Genomic analysis revealed a gene annotated as anaerobic dimethyl sulfoxide reductase chain A (IGTS8_peg1476) whose gene product is 77.8% identical (E-value: 0.0) to a putative biotin sulfoxide reductase (BisC, PF00384) from *R. opacus* strain B4. Therefore, it is tempting to propose that IGTS8_peg1476 is a homolog of BisC that catalyzes the reduction of biotin sulfoxide back to

biotin; however, it was not detected in the proteome (Table S1). The proteome also revealed a protein annotated as biotin-protein ligase (IGTS8_peg880) with 86% (E-value: 9.4e-174) and 70% (E-value: 2.5e-134) identity to a bifunctional protein BirA from *Rhodococcus* sp. strain AD45 (UniProtKB-A0A0D8HQE1) and BirA family transcriptional regulator/biotin operon repressor from *Rhodococcus triatomae* (UniProtKB-A0A1G8FEA0), respectively. Both proteins catalyze the biotinylation of a lysine residue in biotin carboxyl-carrier protein as a component of the acetyl-CoA carboxylase complex (Table S1).

Discussion

Fate of sulfite in the biodesulfurizing culture

A key finding of our study is the increased biosynthesis of components of the sulfate activation complex (6, 7) in the biodesulfurizing culture. Although upregulation of the sulfate activation complex under sulfate limitation and during the stationary phase has been observed in other bacteria (8, 9), at first glance this seemed an intriguing finding which raised many more questions than what it answered. For instance; what is the role of a sulfate activation complex in the dibenzothiophene culture? Why the dibenzothiophene-grown cells upregulate the sulfate activation complex while other sulfate activation enzymes are already in place? Does the sulfate activation complex in the dibenzothiophene culture catalyze sulfate activation?

Our data suggest alternative routes for sulfite oxidation in *R. qingshengii* IGTS8 corroborated by evidences with varying degrees of strength. One possibility could be oxidation of sulfite to sulfate by a putative sulfite oxidase/oxidoreductase ortholog IGTS8_peg2618 and, in case the active site of this enzyme resides in the cytoplasm, sulfate could be exported by the putative sulfate exporter IGTS8_peg446. Another possibility would be export of sulfite and its spontaneous oxidation outside of the cell under aerobic conditions (10), albeit information about the rate of this chemical reaction under the applied growth conditions is not available. Accordingly, excretion and reimport of sulfate for assimilation, as assumed by Aggarwal et al. (11), would be possible and such a scenario would also explain the reported detection of sulfate in the growth medium of some biodesulfurizing cultures (11).

The role of 1-aminocyclopropane-1-carboxylic acid

Despite the recent findings that ethylene production and sulfur metabolism in plants were shown to have a sort of crosstalk (12), the exclusive detection of 1-aminocyclopropane-1-carboxylic acid (ACC) in the dibenzothiophene culture was intriguing, particularly because homologs of the ACC synthase that produces this metabolite from S-adenosylmethionine (13, 14) could not be identified in the *R. qingshengii* IGTS8 genome. These findings question how ACC was produced and what role it could play under biodesulfurization conditions. ACC synthases are found in plants, however, Xu et al. (15) reported the first bacterial ACC synthase in *Streptomyces* sp. CB01883. Whether ACC is transformed to ethylene in the IGTS8 strain, as it is the case in plants, awaits further investigations since genes encoding homologs of ACC oxidase are lacking in the IGTS8 genome (13, 14). Indeed, plant-associated microbes produce trace amounts of ethylene, albeit via other pathways different from those of plants, and the IGTS8 genome does not encode the bacterial ethylene-forming enzyme (16). Supposing that it is eventually transformed to ethylene, what could be the role of ethylene under biodesulfurization conditions? Ethylene is known in plants as a regulator of growth and development in addition to its role in defense response to biotic and abiotic stress (13, 14). In addition, ACC functions as a signaling molecule (12). As the data that we have do not provide clear answers, we can only speculate at this stage. ACC might be produced from S-adenosylmethionine and oxidized to ethylene via yet unknown pathways. Alternatively, ACC may be incorporated into some natural products (5). We might also consider that ACC is a byproduct of S-adenosylmethionine cleavage, while the key product is 5'-methylthioadenosine which was also more abundant in the dibenzothiophene culture (15).

References

1. Cregut M, Durand MJ, Thouand G. 2014. The Diversity and Functions of Choline Sulphatases in Microorganisms. Microb Ecol 67:350–357. doi: 10.1007/s00248-013-0328-7.

2. Czechowska K, Reimmann C, van der Meer JR. 2013. Characterization of a MexAB-OprM efflux system necessary for productive metabolism of pseudomonas azelaica HBP1 on 2-hydroxybiphenyl. Front Microbiol 4: 203. doi: 10.3389/fmicb.2013.00203.
3. Niehaus TD, Folz J, McCarty DR, Cooper AJL, Amador DM, Fiehn O, Hanson AD. 2018. Identification of a metabolic disposal route for the oncometabolite S-(2-

succino)cysteine in Bacillus subtilis. J Biol Chem 293:8255–8263. doi: 10.1074/jbc.RA118.002925.
4. Schwarz G, Mendel RR, Ribbe MW 2009. Molybdenum cofactors, enzymes and pathways. Nature 460(7257):839-47. doi: 10.1038/nature08302.
5. Lin S, Cronan JE 2011. Closing in on complete pathways of biotin biosynthesis. Mol Biosyst 7(6): 1811-21. doi: 10.1039/c1mb05022b.
6. Pinto R, Harrison JS, Hsu T, Jacobs WR, Leyh TS. 2007. Sulfite reduction in mycobacteria. J Bacteriol 189:6714–6722. doi: 10.1128/JB.00487-07.
7. Poyraz Ö, Brunner K, Lohkamp B, Axelsson H, Hammarström LGJ, Schnell R, Schneider G. 2015. Crystal structures of the kinase domain of the sulfate-activating complex in Mycobacterium tuberculosis. PLoS One 10: e0121494. doi: 10.1371/journal.pone.0121494.
8. Hatzios SK, Bertozzi CR. 2011. The regulation of sulfur metabolism in mycobacterium tuberculosis. PLoS Pathog. 7(7): e1002036. doi: 10.1371/journal.ppat.
9. Paritala H, Carroll K. 2013. New targets and inhibitors of mycobacterial sulfur metabolism. Infect Disord - Drug Targets 13:85–115. doi: 10.2174/18715265113139990022.
10. Hui PK, Palmer HJ. 1991. Uncatalyzed oxidation of aqueous sodium sulfite and its ability to simulate bacterial respiration. Biotechnol Bioeng 37(4): 392-6. doi: 10.1002/bit.260370416.
11. Aggarwal S, Karimi IA, Kilbane JJ, Lee DY. 2012. Roles of sulfite oxidoreductase and sulfite reductase in improving desulfurization by *Rhodococcus erythropolis*. Mol Biosyst 8:2724–2732. doi: 10.1039/c2mb25127b.
12. Wawrzynska A, Moniuszko G, Sirko A. 2015. Links between ethylene and sulfur nutrition-a regulatory interplay or just metabolite association? Front Plant Sci 6:1053. doi: 10.3389/fpls.2015.01053.
13. Nascimento FX, Rossi MJ, Glick BR. 2018. Ethylene and 1-aminocyclopropane-1-carboxylate (ACC) in plant-bacterial interactions. Front Plant Sci 9: 114. doi: 10.3389/fpls.2018.00114.
14. Fernández-Llamosas H, Ibero J, Thijs S, Imperato V, Vangronsveld J, Díaz E, Carmona M. 2020. Enhancing the rice seedlings growth promotion abilities of azoarcus sp. Cib by heterologous expression of acc deaminase to improve performance of plants exposed to cadmium stress. 8(9):1453. doi: 10.3390/microorganisms8091453.
15. Xu Z, Pan G, Zhou H, Shen B. 2018. Discovery and characterization of 1-aminocyclopropane-1-carboxylic acid synthase of bacterial origin. J Am Chem Soc 140(49): 16957-16961.doi: 10.1021/jacs.8b11463.
16. Eckert C, Xu W, Xiong W, Lynch S, Ungerer J, Tao L, Gill R, Maness PC, Yu J. 2014. Ethylene-forming enzyme and bioethylene production. Biotechnol Biofuels 3:7(1):33.doi: 10.1186/1754-6834-7-33.
17. Kanehisa M, Sato Y, Morishima K. 2016. BlastKOALA and GhostKOALA: KEGG tools for functional characterization of genome and metagenome sequences. J Mol Biol 428:726–731. doi: 10.1016/j.jmb.2015.11.006.

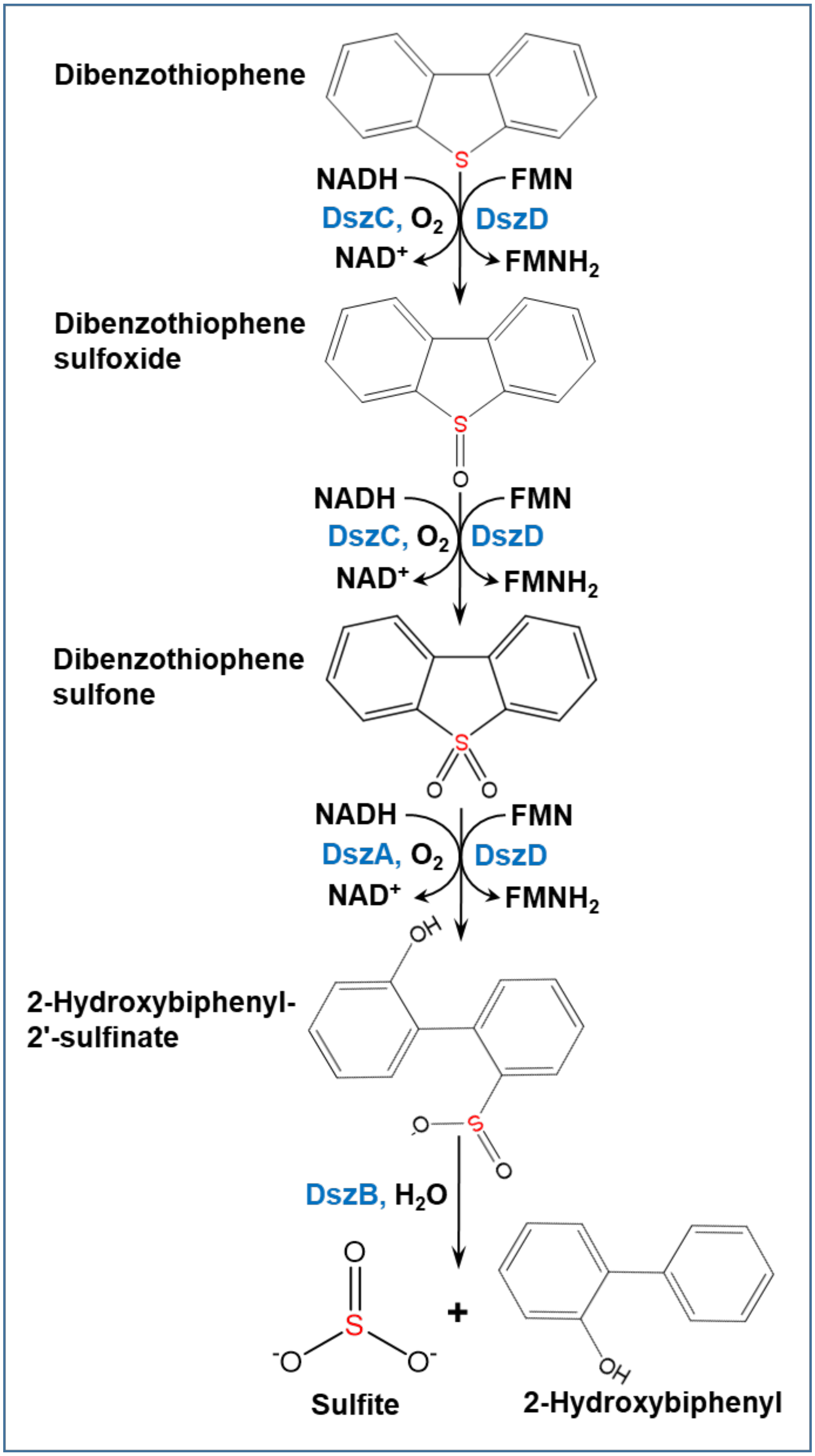

FIG S1 The 4S biodesulfurization pathway.

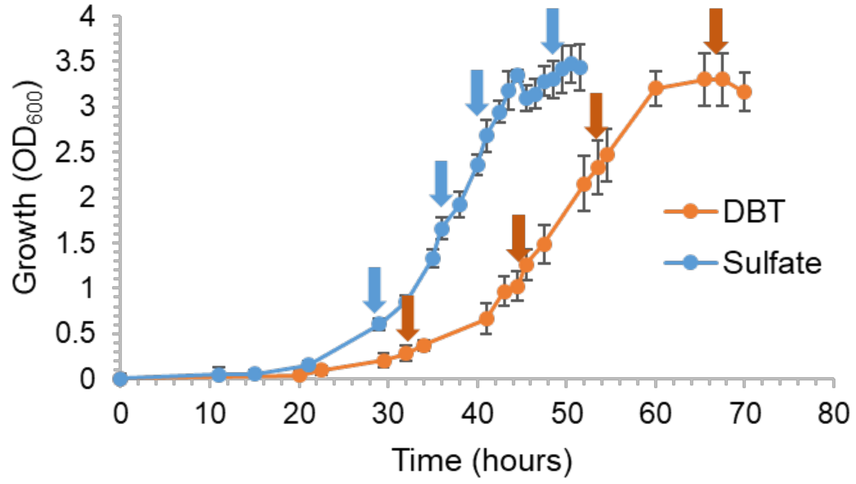

FIG S2 Growth profile of the IGTS8 strain in chemically defined medium with 0.5 mM of either DBT (dibenzothiophene) or $MgSO_4$ as the sole sulfur source and 20 mM glucose as the sole carbon source. Cells were harvested by centrifugation at time intervals as indicated by the arrows.

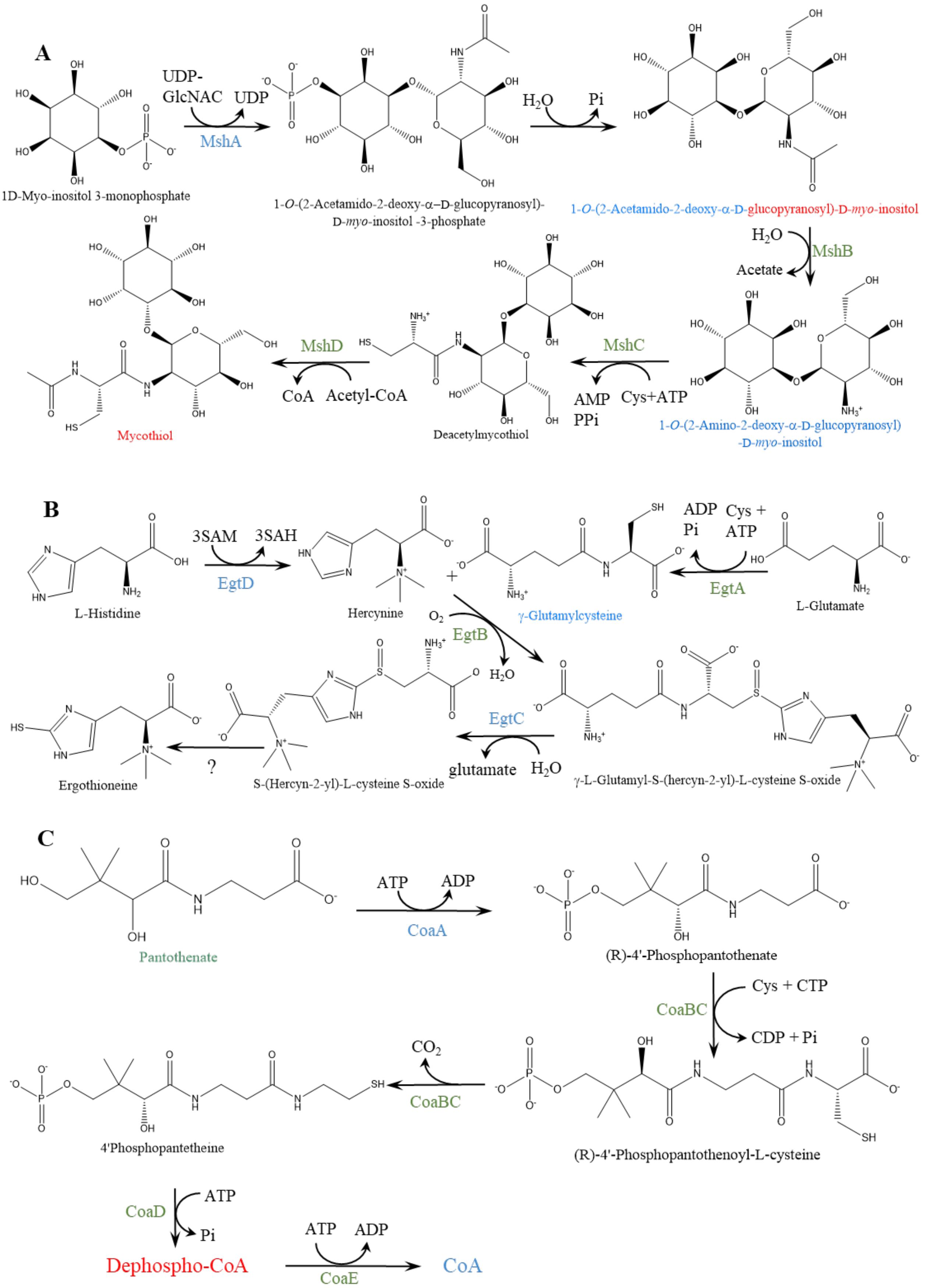
A
UDP-GlcNAC
UDP
MshA
1D-Myo-inositol 3-monophosphate
1-O-(2-Acetamido-2-deoxy-α–D-glucopyranosyl)-D-myo-inositol -3-phosphate
H2O
Pi
1-O-(2-Acetamido-2-deoxy-α-D-glucopyranosyl)-D-myo-inositol
H2O
MshB
Acetate
1-O-(2-Amino-2-deoxy-α-D-glucopyranosyl)-D-myo-inositol
MshC
AMP PPi
Cys+ATP
Deacetylmycothiol
MshD
CoA
Acetyl-CoA
Mycothiol
B
3SAM
3SAH
EgtD
L-Histidine
Hercynine
γ-Glutamylcysteine
ADP Pi
Cys + ATP
EgtA
L-Glutamate
O2
EgtB
H2O
γ-L-Glutamyl-S-(hercyn-2-yl)-L-cysteine S-oxide
EgtC
glutamate
H2O
S-(Hercyn-2-yl)-L-cysteine S-oxide
?
Ergothioneine
C
Pantothenate
ATP
ADP
CoaA
(R)-4'-Phosphopantothenate
Cys + CTP
CoaBC
CDP + Pi
(R)-4'-Phosphopantothenoyl-L-cysteine
CO2
CoaBC
4'Phosphopantetheine
CoaD
ATP
Pi
Dephospho-CoA
ATP
ADP
CoaE
CoA

FIG S3 Biosynthesis of mycothiol (A), ergothioneine (B) and CoA (C) in *R. qingshengii* IGTS8. Proteins and metabolites in blue font are significantly more abundant in the dibenzothiophene culture at least during one growth phase, while those appearing in red font are significantly more abundant in the inorganic sulfate culture at least during one growth phase. The abundance of proteins and metabolites appearing in green font was not significantly different between the dibenzothiophene and inorganic sulfate cultures. The substrate of MshB appears in mixed font color because it was more abundant in the dibenzothiophene culture during the early-log and stationary phase, albeit during the mid-log and late-log phases it was more abundant in the inorganic sulfate culture. See Tables S1 and S3 for details of the abundance profile for proteins and metabolites. UDP-GlcNAC: UDP-N-acetyl-glucosamine; cys: cysteine; SAM: S-adenosylmethionine; SAH: S-adenosylhomocysteine.

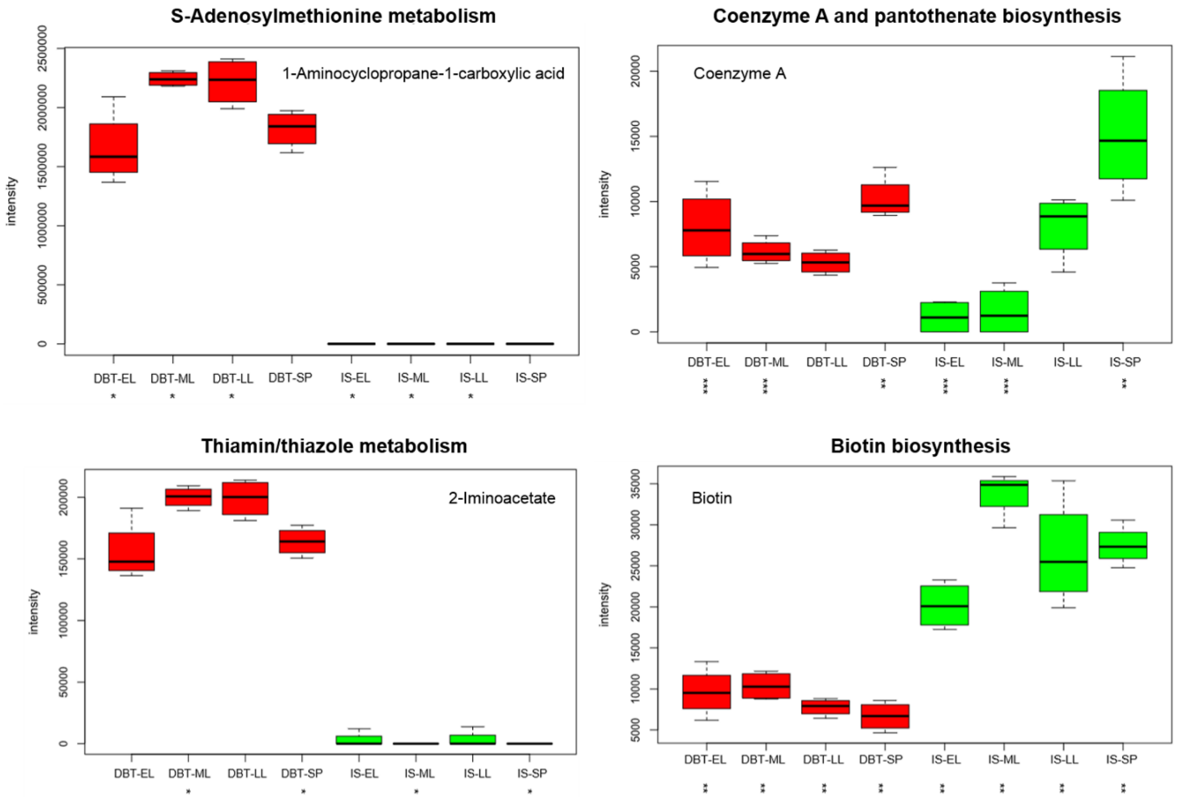

FIG S4 Abundance profile of metabolites of S-adenosylmethionine metabolism and biosynthesis of coenzyme A, pantothenate, thiamin and biotin in the DBT (dibenzothiophene) and IS (inorganic sulfate) cultures from different growth phases. The boxplots indicate the distribution for each growth phase with the minimum, maximum and median values. Red boxplots represent the dibenzothiophene culture and green boxplots represent the inorganic sulfate cultures. The growth phases are abbreviated as EL (earl-log), ML (mid-log), LL (late-log) and SP (stationary phase). Significance of the data [p-value (rank), Wilcoxon test] is indicated by asterisks below the plots: * for $p < 0.01$, ** for $p < 0.05$, *** for $p < 0.1$, no asterisk for $p > 0.1$.

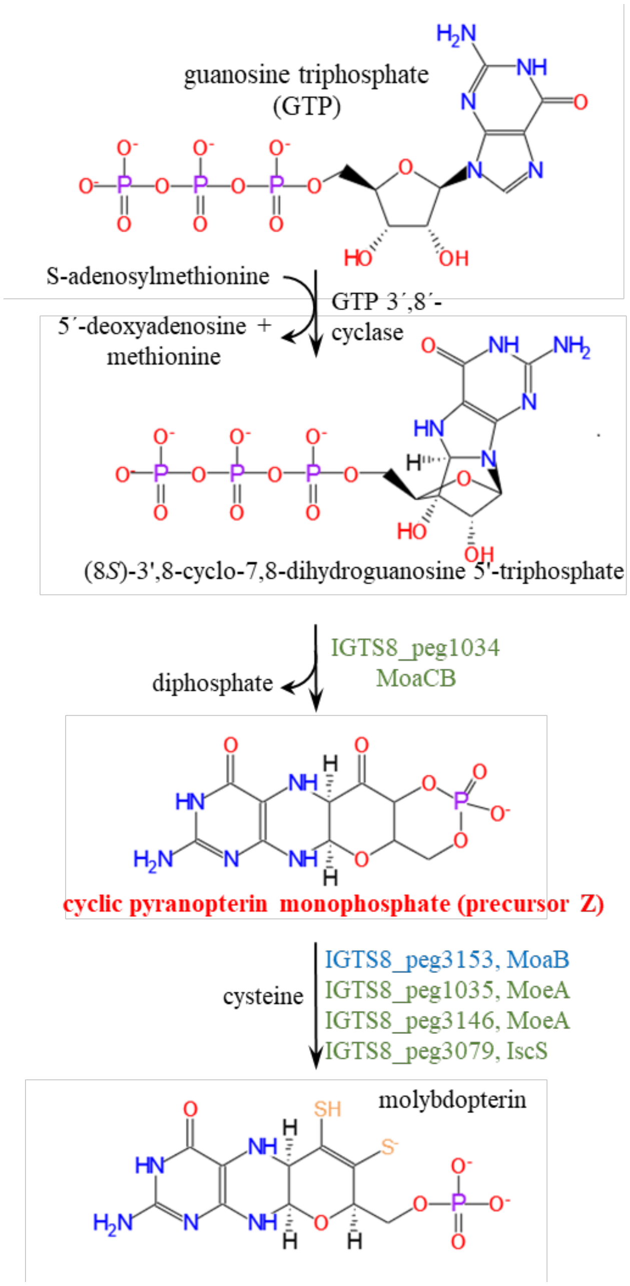

FIG S5 A part of the molybdopterin biosynthesis pathway in *R. qingshengii* IGTS8 showing the proteins and metabolites reported in this study. The chemical structures were copied from the MetaCyc database (https://metacyc.org/META/NEW-IMAGE?type=PATHWAY&object=PWY-6823&detail-level=4) where all the details of the pathway are displayed. Proteins and metabolites in blue font are significantly more abundant in the dibenzothiophene culture at least during one growth phase, while those appearing in red font are significantly more abundant in the inorganic sulfate culture at least during one growth phase. The abundance of proteins appearing in green font was not significantly different between the dibenzothiophene and inorganic sulfate cultures. See Tables S1 and S3 for details of the protein and metabolite abundance profile and proposed functions.

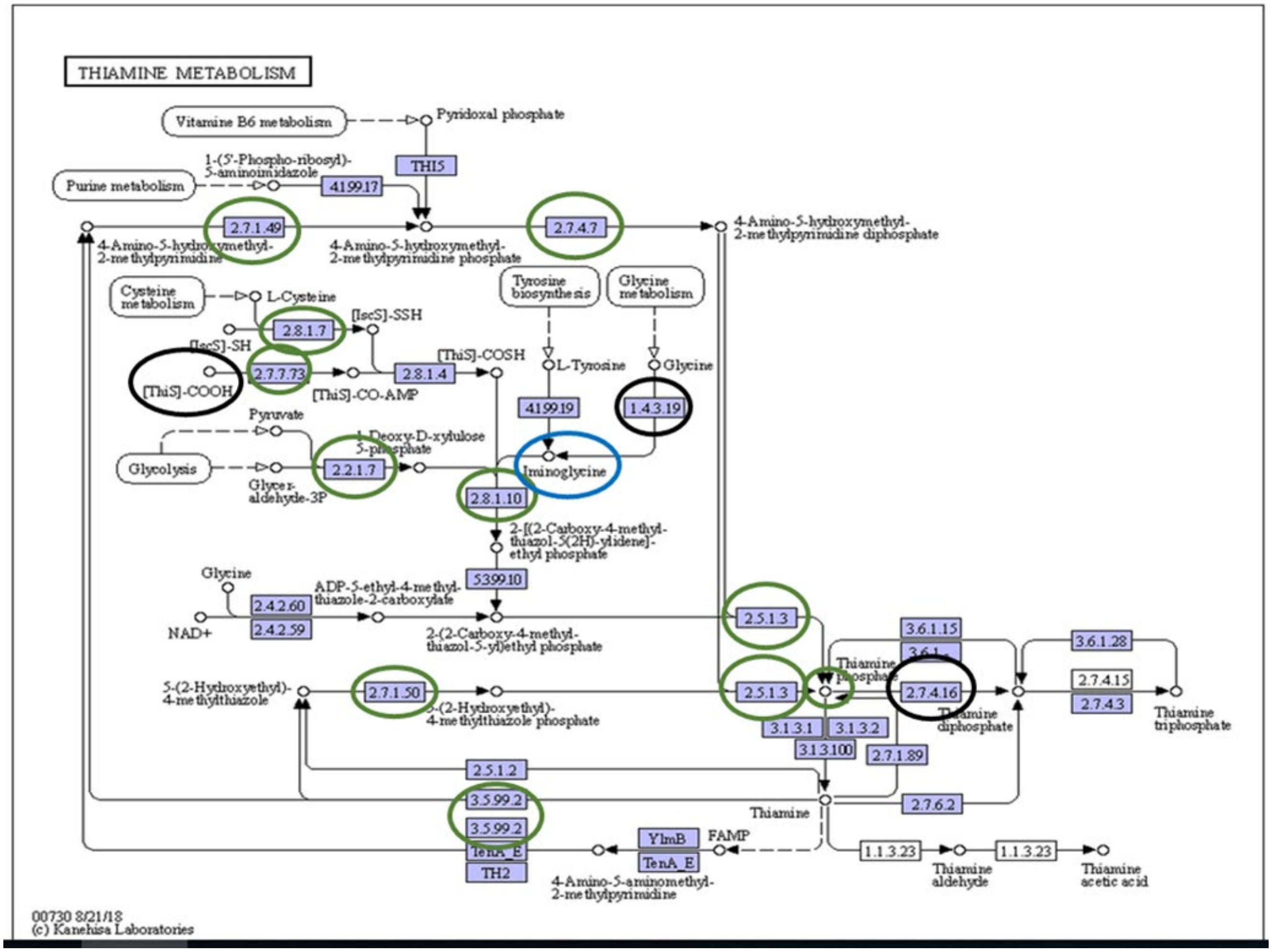

FIG S6 A KEGG map (00730) of thiamin metabolism displaying the enzymes and metabolites of thiamin metabolism in *R. qingshengii* IGTS8. Metabolites within blue circles are significantly more abundant in the dibenzothiophene culture. The abundance of proteins and metabolites appearing within green circles was not significantly different between the dibenzothiophene and inorganic sulfate cultures. In black circles are proteins that were either not detected or detected but could not be quantified. See Tables S1 and S3 for details of the protein and metabolite abundance profile and proposed functions (Reproduced with permission from Kanehisa Laboratories, 17).

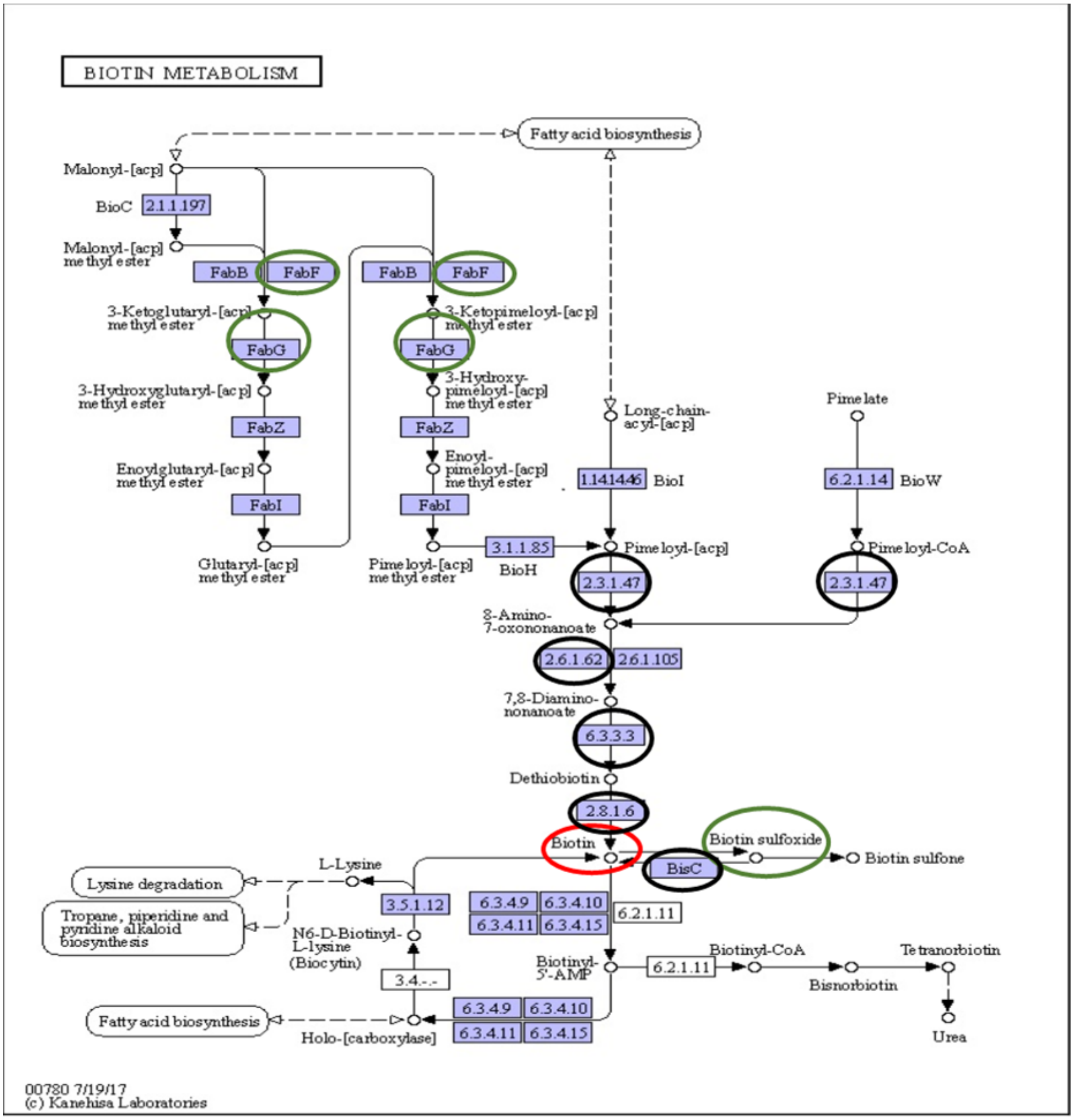

FIG S7 A KEGG map (ko00780) of biotin metabolism displaying the enzymes and metabolites of biotin metabolism in *R. qingshengii* IGTS8. Biotin, in a red circle, was significantly more abundant in the inorganic sulfate culture. The abundance of proteins and metabolites appearing within green circles was not significantly different between the dibenzothiophene and inorganic sulfate cultures. In black circles are proteins that were either not detected or detected but could not be quantified. See Tables S1 and S3 for details of the protein and metabolite abundance profile and proposed functions (Reproduced with permission from Kanehisa Laboratories, 17).

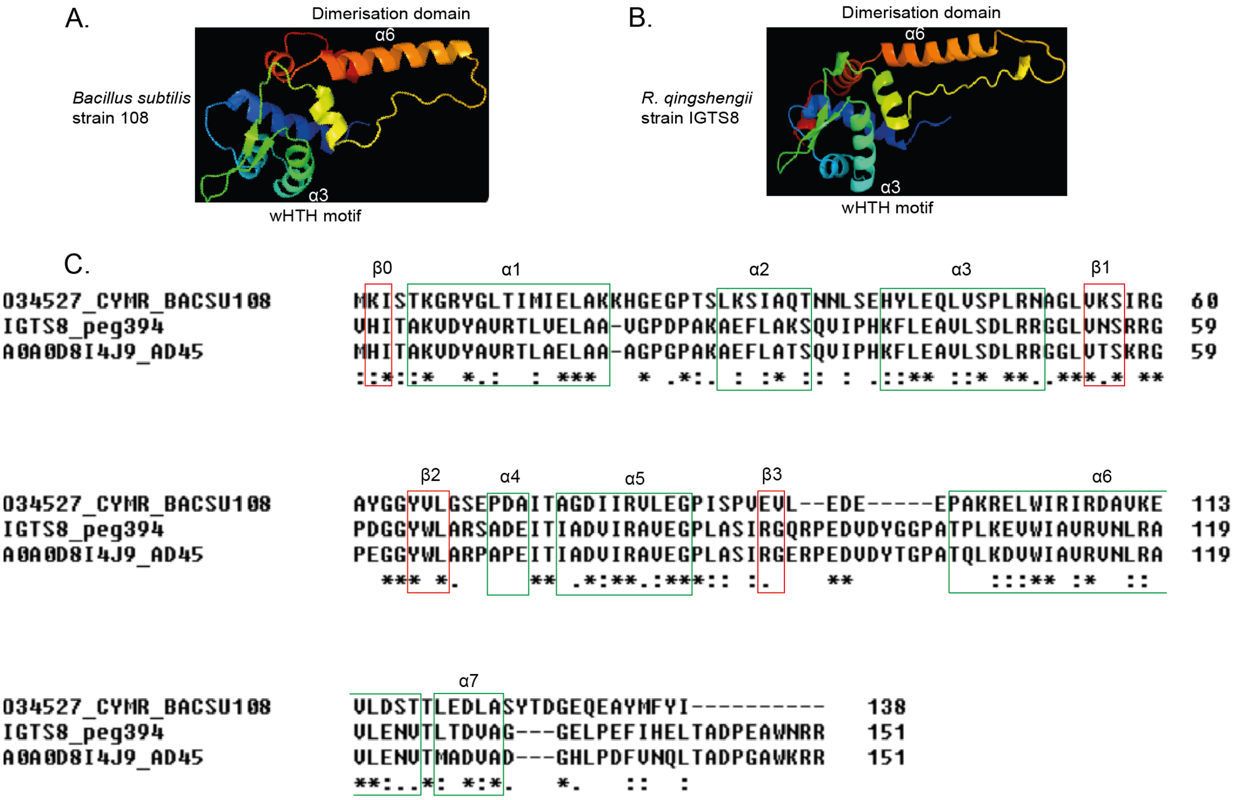

FIG S8 The predicted 3D structure of CymR protein from A. *Bacillus subtilis* strain 108 and B. *R. qingshengii* IGTS8. C. Sequence alignment of CymR proteins from *B. subtilis* strain 108, *Rhodococcus* sp. strain AD45 and strain IGTS8.

Sequences of some differentially expressed proteins showing number of cysteine and methionine residues (See Supplementary Table S1 for the protein abundance)

>IGTS8_peg6007 Cysteine synthase (CysK)
VTTGSLDVDRRTSRQWVDNAVRLIEADAQRSADTHLLRYPLPSNWTVQLYLKDESTHITG
SLKHRLARSLFLYAICNGWVTEETTVIEASSGSTAVSEAYFAKLLGLDFVAVMPASTSPA
KIALIEAQGGRCHFVSDPSSIYSESHRLASELNGHYMDQFTHAERATDWRGNNNIAESIF
TQLEHEEHPIPDWVVVGAGTGGTGATIGRFLRYRKFDTGLCVVDPEHSVFFDAWRASDPS
IVGTRGSRIEGIGRPRVEPSFVGQVVDRMIKVPDAGSIAAARHASAALGRKVGGSTGTNL
WGAFALIAEMMAAGRSGSVVTLLCDGGERYSGTYYNDEWVASEGIDLLGPTAVLDEFVRS
GAWTASS

>IGTS8_peg5907 5-methyltetrahydropteroyltriglutamate-homocysteine methyltransferase (MetE)
MSSSTAAFGSSVLGYPRIGPRRELKRALESYWHGTSDKDALLAVAKELQESTWSELAATG
LSQVPGNTFSFYDHVLDNALLFGAVPERFKPLEGALEPLDFYFTMARGRPDFPPLELVRF
FGTNYYYRQPEIDANTVFSLNSDALLDEFERAKARGIELRPVIMGPVSLLLLSKVGPATE
KSDPNFTPLDLLDALLPEYEKLFEQLAKAGATCVQLDEPSVTEDRTPEELAALGRAYEKL
STAPLRPRLLVTGPYGKFGEALNILAPTKIEAFGLDLVHGKITAEELAAVPNIKRKRIYA
GVVDGRNVWRVDRFNTLTYLNELKDVVPDLVVSTSSSLLHVPYDVLSEYDIPGDVADRLA
FAKQKVGEVVSLAKALTEGPSDKWRKRPTKVHFKLKHAVRARVNAIKPEDRVRAPYEERR
KAQQERLNLPLVPAQTLGSFPQTDAIRQARYELGQGRLEWDDYYKRIQEEIERTIRLQED
IGLDVFVHGEHERNDMVQYFAELLEGYAFTHNGWVQAYGSRCTRPPILYGDIARPKPMTV
EWIAYAQSLTDKPVKGMLTGPVTMIARSFVRQDQPLYETAEQLALVIRDEIADLEAAGIA
IIQVDEPAIRELLPLREDGREAYLEWAVDAFRLATGGAKPETQIHTHLTYSARSSVVDAI
ERLDADVTAIVATRSITWVLEALKEGALTHGVGPGVYESRSARIPDIDELDELLTEASES
ISLDRLWANPDGGLKTRHPWQLEPSLRNMVAAARRLRRRAEQAERDAG

>IGTS8_peg1786 5-methyltetrahydrofolate-homocysteine methyltransferase (MetH)
MLQAADLTLDDFLGLEGCNEILNDTRPDVLKDIHRAYFAAGADAVETNTFGCNLPNLADY
DISHRIRELAEKGTALAREVADEMGPGRDGMARFVLGSMGPGTKLPSLGHAPYAVLRDAY
TEAALGMIDGGADAILVETCQDLLQVKAAILGSQRAMEQLGLRLPIITHVTVETTGTMLL
GSEIGAALTALEPLGIDMIGLNCATGPAEMSEHLRHLSKYSTLPVSVMPNAGLPQLGPKG
AEYPLTADELAEALSGFVSEFGLGLVGGCCGTTPEHISAVADAVRQVEKAQRTPIHEPST
SSLYSAVPFEQDASILMIGERTNSNGSKAFREAMLSADYQKCLDIAKDQTRDGAHMLDLN
VDYVGRDGAADMSELASRLATSSTLPIMLDSTEPAVLQAGLEHLGGRCAVNSVNYEDGAG
PESRFHKIMTLVKEHGAAVVALTIDEEGQARTAETKVAIAKRLIEDIKGNWGLAESDIIV
DALTFPISTGQEEVRRDGIETIEAIRQLKAAYPELHFTLGISNISFGLNPAARQVLNSVF
LHECTEAGLDTAIVHASKILPMARIPDEQRETALDLVYDRRSEGYDPLQKLMALFEGVSA
ASARESRAQELAGLPLFDRLERRIVDGERNGLDDDLTEAMTIKPPLAIINETLLSGMKTV
GELFGSGQMQLPFVLQSAEVMKAAVAFLEPHMEASDEDGKGRIVIATVKGDVHDIGKNLV
DIILSNNGYEVVNIGIKQPIATILDVALDKKADVIGMSGLLVKSTVVMKENLQELNARGV
AEKFPVLLGGAALTRSYVENDLQEVYEGDVSYARDAFEGLNLMDQIMTTKRGGGPAPDSP
EAIAAREKAAERKARHERSKRIAEKRKAAAVPVVLPERSDVATDIAVPTPPFWGSRIVKG
ISLSEYSGLLDERALFLGQWGLRGQRKGDGPTYEELVESEGRPRLRYWLDRLTSEGILAH
AAVVYGYFPAISEGDDVIVLTEPKPDAPERFRFTYPRQHRDRFLCIADFVRSRKDAEATG
QVDVLPFQLVTMGQPIADFANELFASNSYRDYLEVHGIGVQLTESLAEYWHRRVREELVL
PEGHTVAEQDPTEVAGYFDLEYRGARYSFGYGACPDLEDRAKLVALLEPERIGVELSEEL
QLHPEQSTDAFVLHHPEAKYFNV

>IGTS8_peg3888 O-acetylhomoserine sulfhydrylase (EC 2.5.1.49) / O-succinylhomoserine sulfhydrylase (MetY)
MSENTPVDTDPTADWSFETKQVHAGQGPDSATNARALPIYQTTSYVFDNTDHAAALFGLA

EPGNIYTRIMNPTQDAVEQRIAALEGGVAALLVASGQAAESFAILNLAEAGDHIVSSPRL
YGGTYNLFHYTLPKLGITVDFVEDPDNLDHWREAIRPNTKAFYGETISNPKNDVFDIPGI
SAVAHEHGIPLIVDNTVATPYLIQPLKHGADIVVHSATKYLGGHGTAIAGVIVDGGTFDW
TQGRHTNFTTPDPSYHGVTFADLGAPAYALKARVQLLRDLGSAIAPFNAFLISQGIETLS
LRIERHVSNAQKVAEFLEGRAEVTSVAYAGLESSPWHERGKQLAPKGTGAIVAFELAGGV
DAGKKFVNALTLHSHVANIGDVRSLVIHPASTTHSQLTPEEQLASGVTPGLVRLAVGIEG
IDDILADLETGLAAAAS

>IGTS8_peg5771 O-acetylhomoserine sulfhydrylase (EC 2.5.1.49)/O-succinylhomoserine sulfhydrylase (MetZ)
VSGIPQGGAFQRALPDDVGLGTLGVRGGLMRTGFEETSEALFLNSGFVYESAEAAEQAFT
GEVDHFVYSRYGNPTVKMFEERLRLIEGAEACFATASGMSAVFTALGALLANGDRLVAAR
SLFGSCFVVCNEILPRWGVEVVFVDGEDNAQWEEALSVPTTAVFFETPSNPMQSLVDVRR
VSELAHAAGAKVLLDNVFATPLLQRSLDLGADVVVYSGTKHIDGQGRVLGGAILGTEEYI
SGPVRELMRHTGPALSPFNAWTLLKGLETMPVRIRHSVASALEIAEFLEGHSGVKWVKYP
YLTSHPQHELAKSQMSGGGTVVTFELDAPDGEGKKRAFEVLNGLRVIDISNNLGDSKSLI
THPATTTHRAMGPEGRAAVGLTDGVVRLSVGLEDTADLLADLKRALG

>IGTS8_peg5353 Cysteine synthase (EC 2.5.1.47)(CysK1)
MSGIYNNVTELVGRTPIVRLNRLTEGLDAQVAVKLEFYNPANSVKDRIGVAIIDAAEKSG
ALKPGGTIVEGTSGNTGIALAMVGAARGYKVILTMPETMSTERRVMLRAYGAEIVLTPGS
EGMAGAVAKAKEIVEQTENAVSASQFANPANPAIHEATTGEEVWADTDGAVDIFVSGIGT
GGTITGVGRTLRKYKPEVKIVGVEPIDSPILNGGQPGPHKIQGIGANFVPDVLDREIYDE
IIDVSFPDSIEVARALGTKEGILGGISSGAIVWAALELAKRPENAGKLIVAVVCDFGERY
ISTPLFEHIRD

>IGTS8_peg3011 Cystathionine gamma-lyase (EC 4.4.1.1)(MetB)
MSEQRSKADNISWQGFSTKAVHAGYEPDPLTGAVNVPIYASSTFAQDGVGGMRSGFEYAR
TGNPTRRPLEANLAALESGTYGRAFSSGMAATDCLLRSVLRPGDHLVIPDDAYGGTFRLI
DKVFTQWGIEYTPAPVSDVDAVRAAMKSNTKLVWVETPTNPLLNIGDIELLAEVAHEGNA
KIVVDNTFASPYLQQPLQLGADIALHSTTKYIGGHSDVVGGALVCNDEELDTAFAFLQNG
SGGVPGPFDAFLTLRGIKTLALRMERHSDNAEKVVELLDGHPAVAGVIYPGLASHPSHKV
AAKQMRRFGGMISVRLKGGKAAALDLCSRTEIFTLAESLGGVESLIEFPGAMTHASTAGS
ALEVPDDLVRLSVGIEDASDLVGDIEQALS

>IGTS8_peg3012 Cystathionine beta-synthase (EC 4.2.1.22) (CBS)
MRIAEHVVDLIGNTPLVKLTSVTGENFGTVAAKVEYLNPGGSSKDRIAVKMIDAAEASGE
LKPGGTIVEPTSGNTGIGLALVAQKRGYKCVFVCPDKVSEDKRNVLRAYGAEVVVCPTAV
APDNPNSYYSVSDRLTREIPGAWKPNQYSNPGGPESHYETTGPEIWADTDGKITHFVAGV
GTGGTITGTGRYLKEVSGGKVKVIGADPEGSVYSGGTGRPYLVEGVGEDFWPSAYDPSIP
DEIIAVSDADSFEMTRRLAREEGLLVGGSCGMAVVAALEVAKREGPDALIVVLLPDGGRG
YLSKIFNDQWMASYGFLRTPLDGKTNVPTVGDVLRGKSGELPDLVHTHPSETLRDAIEIL
REYGVSQMPVVGAEPPVMAGEVAGSVSERDLLSAVFEGRAHLADSVEKHMSKPFPLIGSG
EPVSAATKALGDTDALMVVDDGKPVGVITRHDLLGFLSSDS

>IGTS8_peg1115 Cystathionine gamma-lyase (EC 4.4.1.1) (CTH)
MSEIGVGRFEDYGDSTRSVSAVAREHAAGQPMQLGPIFAAPYQLSADEGSESDTYARASN
PGWRGLESALAALERADHALVMGSGMSAITATLRSVLVAGDTVVVPSDGYYQVRLYANER
LAPLGITVREVSTLQMGDVSFVDLLDASPGRVVVVAETPSNPVLDVVDLRVLADVCHRSG
ALLIVDNTTATPLGQRPLELGADVVVASGTKALSGHSDLLMGYVATSDAAVSRRIERERL
LSGTVLGPFETWLAHRSLGTAGLRFGRQCDNALALAEMLVNHKAVRSVRYPGLRGDPSYA
TASGQMNRFGGLVSVELDSAQAFHSFVEASALVTAATSFGGIHTTADRRARWGDPVSAGF
VRIACGIEDTADLLADIERALDA

>IGTS8_peg2383 Ferredoxin--sulfite reductase, actinobacterial type (EC 1.8.7.1) (Sir)
MTSTTDAATPADSAAAAEARPARAARPAKRRAEGQWALGYREPLNANEQAKKDDNPLNVR
ARIENIYSKQGFDSIDKGDLRGRFRWWGLYTQREEGYDGTFTGDENIDLLEAKYFMMRVR
CDAGALNVEQLRTIAGISKEFGRNTADLSDRENVQFHWIEVENVPEIWKRLEAVGLKTTE
ACGDCPRVVLGSPLAGESLDEVLDPTPAIDEIVRRYIGDPKYSNLPRKFKTAISGQQDVV
HEINDIAFIGVNHPEHGPGLDLWVGGGLSTNPMLAQRVGAWIPLDEVPDVWEGVVSIFRD
YGYRRLRAKARLKFLVKDWGIEKFREVLETEYLKRPLIDGPAPEKPKRPIDHVGIQKTRN
GHNAVGFAPIAGRVSGDILAQVADAAEKAGSDRIRFTPYQKLIILDVADETLEPLIADLD
ALGLPARPSHWRKNLMACSGIEFCKLSFAETRKRSQVLVPELEQRLADINSQLDVPITIN
INGCPNSCARSQIADIGFKGQLVDDGEGNQVEGFQVHLGGSLGFDSAFGRKLRQHKVTSV
EMGDYIDRVVRQFVKNRNEGERFAEWAVRADEGDLR

>IGTS8_peg2384 Phosphoadenylyl-sulfate reductase [thioredoxin] (EC 1.8.4.8) / Adenylyl-sulfate reductase [thioredoxin] (EC 1.8.4.10) (CysH)
MTVDISRATADELRSIAERGAVELDGASAQELLQWTEDTFGDSYIVASNMQDGVLVHLAA
QVHPGVDVLFLDTGYHFPETIGTRDAVEQVYGVNVINARALASVAEQDVAEGKDLFARDP
NRCCALRKVAPLKQTLSGYSAWVTGIRRVEAPTRANAPLISFDEAFGLVKINPIAPWSDE
EMQNYIDTNSILVNPLVDEGYPSIGCAPCTSKPAPGSDPRSGRWAGASKTECGLHAS

>IGTS8_peg2385 Sulfate adenylyltransferase subunit 2 (EC 2.7.7.4) (CysD)
LTPSRPQLDGTQFDTLDALESEAIHIFREVAGEFERPVILFSGGKDSTVLLHLAIKAFWP
APLPFTLLHVDTGHNLQEVLDFRDHVVAKYNLRLHVASVEEYIADGRLTERPDGIRNPLQ
TVPLLDAISENRFDAVFGGARRDEERARAKERIFSLRNAFGQWDPKKQRPELWNLYNGRH
SPGEQVRVFPLSNFTELDIWRYIARDNVELASIYYAHQRPVYQRDGMWMTPGVWGGPADG
EELQTLSVRYRTVGDGSTTGAVLSDAADNEAILAEVAASRLTERGATRGDDRVSEAAMED
RKREGYF

>IGTS8_peg2386 Sulfate adenylyltransferase subunit 1 (EC 2.7.7.4) (CysN)
MSDLLRLATAGSVDDGKSTLVGRLLYDTKSVLADQIDAVTRASVDRGLDTPDLSLLVDGL
RAEREQGITIDVAYRYFATPKRTFVLADTPGHVQYTRNTVSGASTAQLVILLVDARKGVI
AQTRRHAAVLALLGVPKLVLAVNKIDLVENPEEVFTSISAEFNELTSSLGWASEDVVEIP
VSALHGDNVAIRSDKTPYYTGPTLIEHLESIPVDAEPHRVGLRFPVQYVIRPRTAEFLDY
RGYAGQVAAGSVVPGDEVVILPSGTRTTVTRIDTADGELDVAHAGRSVTLILADDVDVSR
GDTIASPSDAPEPIGEFDATVCWLAEKSLRPGARLLLKHGTRTTQAIVGSLVERFNEQDL
TTDQGPDTLELNEIGKISIRVAEPIVADDYAVNRHTGSFLLIDPAGGNTLAAGLVGDVIF
AVELGDRV

>IGTS8_peg2387 Sirohydrochlorin cobaltochelatase (EC 4.99.1.3) (CbiX-SirB)
MVPLIAVAHGSRDPRSARVIAAAVAALREVRPDLDVRLAFLDLNAPSIDQVLDGVAHDGH
THAVVVPMLLGNAFHARVDLPGLLAAAATRHPQLTVFQADVLGRDARLIEAVRERILEVG
ARPDDPSVGVALAAVGSSDARANAATAELASTLLGGTNWSGVRICFATSAEPTVAQAISA
LEQDGAERVVVAPWFLAPGLLTDRLSAAAATACPRARFADTIGGHSLLIETMIDRYRQVA
DALPGRLVRSA

>IGTS8_peg167 probable sulfatase
VSAARDNVLIIHWHDLGRHLASYGVAGVNSPHLDRLAAEGIRFTDAHATAPLCSPSRGSL
FSGRYPHNNGLIGLAHHGWEYHAEVQTLPQILSDHGWYTSLFGMQHETAYPKRLGYHEYD
VTNSFCDYVVPRAQNWLRRFPPEPFLLTTGFFETHRPYPADRYTPADTAALEVPGYLPDV
PQVREDLAEFHGSVEVADAAVGELLATLEEEGLDENTWVFFLTDHGAAFPRAKSTLYAPG
TGIALIARPPARYRSGGKEYETLFSGVDLVPTILDLLGIDVPTEVEGRSHADALVDRGTE
PSEPVRTSLFTEKTFHDSFDPIRAVRTKDYSYIENYAVRPALDLPLDIEDSLSGKALGTE

HLAVRAPRELYDLRADPDEKINLIDRPEVSAIQAELSSQLHTWRAETDDRLPDEALGSAM
AVHRMAAYLEKIGVRPNVRSAYSADRGYLESDTENP

>IGTS8_peg168 Sulfate adenylyltransferase subunit 1 (EC 2.7.7.4)/ Adenylylsulfate kinase (EC 2.7.1.25) (CysNC)
MTTTITRQLLRLATAGSVDDGKSTLIGRLLHDTDSLPTDHLDSVTDEHGVADLAALSDGL
RAEREQGITIDVAYRFFSTPTRSYVLADTPGHERYTRNMFTGASNAHVAVLLVDARTGVL
RQTRRHARIAALLGVPNLVAVVNKIDLVDFDEARFAQVQEELRALARQVGREDVLAIPVS
AKAGDNVVTRSENTAWYEGPTLLEYLEGVELHAPATHIEELRLPVQWVSRPDPQNRRRYT
GRISAGTLSVGDEVVVLPSGSKSTVISLDTLDDNRSTAVAPLSVSVELADDIDVGRGDVL
VSGGAQAHLPVLARELEATICWLSNTPLRAGDRIALKHTSRTVRATVQELHTRLDPETLD
EQDSPVELGLNDIGTVTLRTSSVVVADTYDHNRDSGAFILIDEQSNDTVGAGTILEPRVV
VPGEQTRNDIKWHPSSLERQRRWASTTQRGATIWLTGLPASGKSTVAVAVERALVDAGRT
AYLLDGDNVRHGISDDLGFSPGDRAENIRRVGHLTRLFADAGVVAIASMVSPLRSDRAIA
RALNEAAGLTFLEVAVTTPVEECERRDPKGLYARARAGELKGLTGIDAPYETPEDPDLAF
DTTGADINELVERVIALLEEREAARP

>IGTS8_peg169 Sulfate adenylyltransferase subunit 2 (EC 2.7.7.4) (CysD)
MSTVTDVVDELRVLESEAVHIIREVVAELERPVLLFSAGKDSIVLLRLAEKAFRPSPLPF
PVLHVDTGHNFEEVIEFRDRRIKEGGHTLLVASVQDSIDQGRVQESTETSGSRNRLQTRT
LLDALEAGRFDAAFGGARRDEERARAKERVLSFRDEFGQWDPRAQRAEPWSLYNGRIRRG
ESVRVFPLSNWTELDIWRYIAQEELELPSIYFAHQRRVFERDGMLLAASEFSTDAENAAA
TTEWVRYRTVGDLTITGAVRSHATTIDDVVTEISAATVSERGETRADDRTSVAAMEDRKR
EGYF

>IGTS8_peg170 Sulfotransferase (Stf0)
LLVESLRATGVAGEPEEFFQYLPETSRSPQPRQWFEDVTDESVLGLLAPFHPGTPDTRTS
EQWRTQLLELGRTPNGVWGGKLMWNQTPLLLDRAAGLPWRSGTDLRSALHDTLDHDLQFI
HVYREDVVAQAVSMWRAVQTQVWRDDATPPNLSDGAQYNAVGIAHLVTILGEQERQWKRW
FEEEDISPIEVGFRDLTEDPQSVVAKTLISLGLDGQLAPPPPLRRQSDGRSREWVQRYRI
DAEQNGYPVT

>IGTS8_peg4196 Putative hydrolase in cluster with formaldehyde/S-nitrosomycothiol reductase MscR
VTLRVDRVVTSGTFSLDGGTWDVDNNIWLIGDDNEVVIVDAAHTAQPIIDAVGGRKVIGI
VCTHGHNDHVTVAPELAEKLDAPIYLNPADDVLWEMTHPGVKHLSLEDDQRIAVAGTDIL
AIHTPGHSPGSTCLYLPEAGELFSGDTLFSGGPGATGRSYSDFPTIIESIRDKLFALPAE
TKVHTGHGDGTTIGTEAPHLEEWIRAGS

>IGTS8_peg1918 Thiosulfate sulfurtransferase, rhodanese (EC 2.8.1.1)) (TrxA)
VATQTLTQQNFDETINGSDVVLVDFWASWCGPCRQFAPTFEASSEKHADVVHAKVDTEAE
QGIAAAANIRSIPTIMAFREGVLVFNQAGALPAAQLEELVTQVKALDMDEVRKQIAEQTA
SAE

>IGTS8_peg751 possible rhodanese-related sulfurtransferase (PspE)
MLEVDLNALDTALAAGEPLIDVREADEFAQVRVPGATLIPLSEFVSRVGEIPDAETVYII
CAVGGRSLQAAEYLQARGINAVSVAGGTMAWYQSGRQVETGE

>IGTS8_peg4995 2-hydroxychromene-2-carboxylate isomerase/DsbA-like thioredoxin domain
VTIEVWSDVACPWCYIGKTRFLSALDRFENKDRVNVIWRSYQLAPETPVGEGRTELDALV

EMKGMAPEQVRQMFAHVSATAAEVGLTLDFDTVIAANTFDAHRLLHLAGERQNELLEALF
KAHFSDGKVIDDREVLVELAVSVGLDADVVREQLGSDAAAEAVREDLSMARQLQVSGVPF
FVANRAVAVSGAQPEEVFLQLLTQASEPA

>IGTS8_peg352 D-3-phosphoglycerate dehydrogenase (EC 1.1.1.95)
MTDASAPRIHLGPVEDPHVADGIRRAGGVLVPLADAEGVVWVHGPETFPSSLPDSVRWVQ
LPFAGIEPWFDAGVIDDKRVWTSAAGAYAGNVAEHTMMLLLAGVRSLPEQLAAQSWRKEE
FDPRVGTLQGSTVAIIGCGGIGRALIPYLAASKVKVIAITRSGTPVEGASETLSADRTGE
IWSKADHFVIAAPSTSATRHLVGKAELDQMSESSWIVNIARGTLVDTDALVDALEAGSIG
GAALDVTDPEPLPDGHRLWKLPNAIITPHVANPATTLTRVLADHVAANVARFAAGEDLAA
VIDPSAGY

>IGTS8_peg5938 Choline-sulfatase (EC 3.1.6.6) (AtsA1)
MTSPDSAPPPHARGYEGFEGRIGRTAADSTPSWPTSTTPIPGSPNIVVVLIDDMGYSDIG
PFGSEIETPTLDRLAAQGIRMTNYHTTPLCSPSRAALLTGLNPHRAGYGFVANADPGYPG
LRLELADDVQTLPEILRGAGYATYAVGKWHLVRDANLAPGRSRDSWPTQRGFDRYYGSLE
GLNSFYYPNQLISDNSVVDVDEYPEGYYLTDDLTDKAVGYIKDLRAHDQDKPFFLYFAHV
AMHGPLQAKPADQEKYRGRYDEGWDRIRESRFASQLVQGLFPEGTQQAPRNSEPGFDVPE
WDSLTPEVQSRFARYMEVYAAMVDSIDQSVGRIVDTLDELGELDNTIIVFTSDNGGTAEG
GSDGTRSYFAQFAHIQDPDWVGDVPHDESLIGGPELGVHYPRGWGQTSNTPFRFYKGQSF
AGGIRVPFLLSWPAGLKPEADDNGIRDQFAYVTDLTPTLLELAGIDAPSVRNGLPVKEFD
GVSSASVLRSSAAPSTHTEQYTEMTGNRGYYKDGWKLLALTPADIDKPHWQLFDVRTDPT
ELDDLASRFPDKVRELAEAWDHSAWANTVFPLLGNTVGAVRRPEEGALSRPVRLLAGTPP
LERYRSSKLIGFRDFDITVELDGYREGDAGVLVAHGDPQGGYLLYVEDGHLHLGYNAFGS
YSVVDAGPLALGTERIGLSVTVVPRLRWDLHVSVDGIHAGQLLGQVQLVGMAPWTGISVG
VDARGPVSWEVRRRHGPFRYSGALRAVTYTPGRIQVPRRHIESIEREAEYAAD

>IGTS8_peg5939 Alpha-ketoglutarate-dependent taurine dioxygenase (EC 1.14.11.17) (TauD)
MSTAFAKPTLTNSASTNGAAVDAARGTVTTLGAHIGARIDGITLGGHLDPATISLIRQAL
LEHKVIFFRGQDHLDNDSQYEFAQLLGTPTTAHPTVKSHGAKVLPIDSDLGKANSWHTDV
TFVDRIPKASILRAVQLPEYGGSTTWASGVAAYNGLPDPLKALAENLWARHTNVYDYAAT
SAERLTEDRTAAYREEFQSTYFETEHPVVRVHPETGERTLVLGHFVKNFVGLSTEQSNGV
FKLLQDHAIKLEYTTRWNWEAGDVAIWDNRATQHYAIADYDDQYRRLERITLAGDIPVNI
HGERSRSIAGDASEYSIIEGASTLP

>pIGTS8_peg6351 dibenzothiophene desulfurization enzyme A (DszA)
MHLAGFFSAGNVTHAHGAWRHTDASNDFLSGKYYQHIARTLERGKFDLLFLPDGLAVEDS
YGDNLDTGVGLGGQGAVALEPASVVATMAAVTEHLGLGATISATYYPPYHVARVFATLDQ
LSGGRVSWNVVTSLNDAEARNFGINQHLEHDARYDRADEFLEAVKKLWNSWDEDALVLDK
AAGVFADPAKVHYVDHHGEWLNVRGPLQVPRSPQGEPVILQAGLSPRGRRFAGKWAEAVF
SLAPNLEVMQATYQGIKAEVDAAGRDPDQTKIFTAVMPVLGESQAVAQERLEYLNSLVHP
EVGLSTLSSHTGINLAAYPLDTPIKDILRDLQDRNVPTQLHMFAAATHSEELTLAEMGRR
YGTNVGFVPQWAGTGEQIADELIRHFEGGAADGFIISPAFLPGSYDEFVDQVVPVLQDRG
YFRTEYQGNTLRDHLGLRVPQLQGQPS

>pIGTS8_peg6352 Dibenzothiophene desulfurization enzyme B (DszB)
MTSRVDPANPGSELDSAIRDTLTYSNCPVPNALLTASESGFLDAAGIELDVLSGQQGTVH
FTYDQPAYTRFGGEIPPLLSEGLRAPGRTRLLGITPLLGRQGFFVRDDSPITAAADLAGR
RIGVSASAIRILRGQLGDYLELDPWRQTLVALGSWEARALLHTLEHGELGVDDVELVPIS
SPGVDVPAEQLEESATVKGADLFPDVARGQAAVLASGDVDALYSWLPWAGELQATGARPV
VDLGLDERNAYASVWTVSSGLVRQRPGLVQRLVDAAVDAGLWARDHSDAVTSLHAANLGV

STGAVGQGFGADFQQRLVPRLDHDALALLERTQQFLLTNNLLQEPVALDQWAAPEFLNNS
LNRHR

>pIGTS8_peg6353 Acyl-CoA dehydrogenase;probable dibenzothiophene desulfurization enzyme (DszC)

MTLSPEKQHVRPRDAADNDPVAVARGLAEKWRATAVERDRAGGSATAEREDLRASGLLSL
LVPREYGGWGADWPTAIEVVREIAAADGSLGHLFGYHLTNAPMIELIGSQEQEEHLYTQI
AQNNWWTGNASSENNSHVLDWKVSATPTEDGGYVLNGTKHFCSGAKGSDLLFVFGVVQDD
SPQQGAIIAAAIPTSRAGVTPNDDWAAIGMRQTDSGSTDFHNVKVEPDEVLGAPNAFVLA
FIQSERGSLFAPIAQLIFANVYLGIAHGALDAAREYTRTQARPWTPAGIQQATEDPYTIR
SYGEFTIALQGADAAAREAAHLLQTVWDKGDALTPEDRGELMVKVSGVKALATNAALNIS
SGVFEVIGARGTHPRYGFDRFWRNVRTHSLHDPVSYKIADVGKHTLNGQYPIPGFTS

>IGTS8_peg4243 Coenzyme F420-dependent N5, N10-methylene tetrahydromethanopterin reductase and related flavin-dependent oxidoreductases; sulfonate monooxygenase (SfnG)

LLGFMSTERIADEIKFAYWVPNVSGGLVTSDIEQRTSWDYEYNKKLAQTAENNGFEYALS
QVRYEASYGAEFQHESTSFSLALLLATEKLKVIAAVHPGLWQPAVLAKLGATADHLSNGR
FAVNVVSGWFKDEFTHLGEPWLEHDERYRRSAEFLQVLRKIWTEDDVDFRGDFYRIHDFT
LKPKPLNTPERPNPELFQGGNSAAARENAGRYSDWYFSNGKDYDGVTEQLVDVRRVAREN
DREVKFGLNGFIIARDTEAEAKDTLREIIAKANRPAVEGFKNAVQQAGASTASKDGMWAD
STFEDLVQYNDGFRSQLIGTPEQIAHRIVEYRRRGVDLILGGFLHFQEEIEYFGAKVLPL
VRELEAADSDEQVLVTAG

>IGTS8_peg5732 Nitrilotriacetate monooxygenase component A (EC 1.14.13.-) (ScmK)

MSDSPRQLSLNAFIHPAGHHEAAWRHPWTTPERLFDVTYFQEIARTAEAAKFDGIFFADG
PALRSDVEHGPAGTLEPITLLTAIAVVTERIGLIATASTTYYEPYNLARLFASLDHISNG
RAGWNIVTTGTDLAAANFGLAKHPDHGDRYSRAREFVDAVVRLWDSWEDDAISLDRENGI
YADRNKIHEINYVGRHLRVRGPFNAPRTPQGYPVLVQAGASNDGRAFAGQYAEAIFTAHQ
RLSDAQAFYTDIKSRAAQFGRNPDHVKILPGISPFIGDTEEQAKALEREFNELTSPEYGL
AQLGALTGTDVRNLELDAPVPVELFAAAGDVTDNKQSRLQVIAGIVERERPTVRGLLHRL
AGARGHRVFAGTAEQVADTIEEWFTSGAADGFNVMPPYYPGGLEIFTERVVPILQDRGLF
RTEYTGTTLRDHFGLPRPESQFSGSASAAQ

>IGTS8_peg3535 Nitrilotriacetate monooxygenase component A (EC 1.14.13.-)

MTASRSAVPRPDAHVHFGVFFQGVNHTTIWSDSASGSQIDFETFRRLVLTAERGLFDAFF
LGEGLRLREQNGKILDLDIAGRPDAIAQLAALAGITDKIGLVATQNTTYNEPADLARRLS
GLDLLSDGRAGWNAVTTDNAWTGENFRRGGFLDHSLRYERAGQFIETARALWDSWADDAI
SDSRTSENWSTPGAVGDVRRQTSQFDISVTPTLPRSRQGHPVIFQAGDSPSGRDFAATHA
DVIFSRHGTHFDGALDFADDIRARLRKAGRPEDDVKILPSTQIVLAENEPEVEEKARWVL
EGQFTGQTALSLVGLVWGKDLSDRDPDGPLPEEDPVARPVSETRGSARDGNDPIAIAREW
RALAEAKNLSLRQVAIETSQRSGFAGTPGQVADQLVHWVRNGASDGFNISPYLVPTGLDE
IVDWLIPELQERGAYRTEYSTSTLREHLGLRPPLTRRTTSEA

Table S1: Sulfur metabolism proteome in *R. qingshengii* IGTS8*

Protein ID	Protein Annotation	Log2 Fold Change#				EC/TC No. /Protein Name	Pfam No.	Pathway/Proposed Function
		EL	ML	LL	SP			
pIGTS8_peg6351	Dibenzothiophene desulfurization enzyme A	8.6	7.9	6.3	8	EC: 1.14.1.22/DszA	PF00296	Sulfur acquisition from DBT (4S pathway)
pIGTS8_peg6352	Dibenzothiophene desulfurization enzyme B	8.3	7.2	6.6	7.8	EC: 3.13.1.3/DszB	PF09084	Sulfur acquisition from DBT (4S pathway)
pIGTS8_peg6353	Acyl-CoA dehydrogenase; probable dibenzothiophene desulfurization enzyme	6.1	5.5	5.3	5.2	EC: 1.14.14.21/DszC	PF08028	Sulfur acquisition from DBT (4S pathway)
IGTS8_peg2794	Nitrilotriacetate monooxygenase component B	3.8	6.8	5.5	D	EC: 1.5.1.42/DszD	PF01613	Flavin reductase (4S pathway)
IGTS8_peg3410	Putative monooxygenase	4.2	3.9	3.1	D	EC: 1.14.14.10	PF00296	Sulfur acquisition
IGTS8_peg3411	Taurine transport system permease protein	D	D	D	D	TC: 3.A.1.17.1/TauC	PF00528	ABC transporter
IGTS8_peg3412	Taurine transport ATP-binding protein	DS	DS	DS	DS	TC: 3.A.1.17.1/TauB	PF00005	ABC transporter
IGTS8_peg3413	Possible ABC sulfonate transporter, substrate binding component	D	D	5.1	4.6	TC: 3.A.1.17.1/TauA	PF09084	ABC transporter
IGTS8_peg3414	RNA polymerase sigma-70 factor	2	2	2.5	2.4	SigJ	PF08281	Transcriptional regulator
IGTS8_peg3624	ABC-type nitrate/sulfonate/bicarbonate transport system, ATPase component	6.1	6.3	6.9	5.8	EC: 7.6.2.7/TauB	PF00005	ABC transporter
IGTS8_peg3625	Taurine-binding periplasmic protein	2.7	2.6	2.5	2.5	TC: 3.A.1.17.1/TauA	PF09084	ABC transporter

Protein ID	Protein Annotation	Log2 Fold Change#				EC/TC No. /Protein Name	Pfam No.	Pathway/Proposed Function
		EL	ML	LL	SP			
IGTS8_peg3626	ABC-type nitrate/sulfonate/bicarbonate transport system, permease component	ND	ND	ND	ND	TC 3.A.1.17.1/TauC	PF00528	ABC transporter
IGTS8_peg4798	Transcriptional regulator, IclR family	ND	ND	ND	ND	--------------------	PF01614	Transcriptional regulator
IGTS8_peg4799	Alkanesulfonates-binding protein	5.8	5.7	5.8	4.1	TC: 3.A.1.17.2/SsuA	PF09084	ABC transporter
IGTS8_peg4800	Alkanesulfonates transport system permease protein	ND	ND	ND	ND	TC: 3.A.1.17.2/SsuC	PF00528	ABC transporter
IGTS8_peg4801	Alkanesulfonates ABC transporter ATP-binding protein	D	D	D	-0.9	TC: 3.A.1.17.2/SsuB	PF00005	ABC transporter
IGTS8_peg4802	Dibenzothiophene desulfurization enzyme B	D	D	D	DS	SsuA/DszB	PF09084	Sulfur acquisition
IGTS8_peg4803	Alkanesulfonate monooxygenase	1.1	D	0.8	D	EC: 1.14.14.5/SsuD	PF00296	Sulfur acquisition
IGTS8_peg4804	Alkanesulfonate monooxygenase	D	DS	DS	D	EC: 1.14.14.5/SsuD	PF00296	Sulfur acquisition
IGTS8_peg4806	Alkanesulfonates-binding protein	7.6	7.9	7.7	8	TC: 3.A.1.17.2/SsuA	PF09084	ABC-transporter
IGTS8_peg3800	Methionine ABC transporter substrate-binding protein	7.1	6.3	5.9	6	TC: 3.A.1.24/MetQ	PF03180	Methionine transport
IGTS8_peg3801	Methionine ABC transporter ATP-binding protein	D	D	3.7	DS	TC: 3.A.1.24/MetN	PF00005	Methionine transport
IGTS8_peg3802	Methionine ABC transporter permease protein	D	D	D	D	TC: 3.A.1.24/MetI	PF00528	Methionine transport
IGTS8_peg2391	Sulfate and thiosulfate import ATP-binding protein	D	3.1	2	D	EC: 3.6.3.25/CysA	PF00005	Sulfate uptake

Protein ID	Protein Annotation	Log2 Fold Change#				EC/TC No. /Protein Name	Pfam No.	Pathway/Proposed Function
		EL	ML	LL	SP			
IGTS8_peg2392	Sulfate transport system permease protein	ND	ND	ND	ND	TC: 3.A.1.6/ CysW	PF00528	Sulfate uptake
IGTS8_peg2393	Sulfate transport system permease protein	ND	ND	ND	ND	TC: 3.A.1.6/ CysT	PF00528	Sulfate uptake
IGTS8_peg2394	Sulfate transport system permease protein	ND	ND	ND	ND	TC: 3.A.1.6/ CysT	PF00528	Sulfate uptake
IGTS8_peg2395	Sulfate and thiosulfate binding protein	1.4	1.4	1.2	0.9	TC: 3.A.1.6/CysP/Sbp	PF13531	Sulfate uptake
IGTS8_peg167	Probable sulfatase	D	D	3.7	3.6	----------------------------	PF00884	Sulfur acquisition
IGTS8_peg168	Sulfate adenylyltransferase subunit1/Adenylylsulfate kinase	D	D	D	D	EC:2.7.7.4/2.7.2.25/CysN2C	PF01583	Assimilatory sulfate reduction
IGTS8_peg169	Sulfate adenylyltransferase subunit 2	DS	DS	DS	DS	EC: 2.7.7.4/CysD2	PF01507	Assimilatory sulfate reduction
IGTS8_peg170	Sulfotransferase	DS	D	D	DS	EC: 2.8.2.37/Stf0	PF09037	Biosynthesis of sulfated metabolites
IGTS8_peg2383	Ferredoxin-sulfite reductase, actinobacterial type	D	D	D	-0.8	EC: 1.8.7.1/CysI	PF01077	Assimilatory sulfate reduction
IGTS8_peg2384	Phosphoadenylyl-sulfate reductase [thioredoxin]/ (Adenylyl-sulfate reductase [thioredoxin]	-0.6	0.6	1	2.1	EC: 1.8.4.8/1.8.4.10/CysH	PF01507	Assimilatory sulfate reduction
IGTS8_peg2385	Sulfate adenylyltransferase subunit 2	D	0.1	0.9	1.2	EC: 2.7.7.4/CysD1	PF01507	Assimilatory sulfate reduction
IGTS8_peg2386	Sulfate adenylyltransferase subunit 1	0.7	0.8	0.6	0.4	EC: 2.7.7.4/CysN1	PF00009	Assimilatory sulfate reduction

Protein ID	Protein Annotation	Log2 Fold Change#				EC/TC No. /Protein Name	Pfam No.	Pathway/Proposed Function
		EL	ML	LL	SP			
IGTS8_peg2387	Sirohydrochlorin cobaltochelatase	ND	ND	ND	ND	EC: 4.99.1.3/CbiX-SirB	PF01903	Cobalamin biosynthesis
IGTS8_peg3888	*O*-acetylhomoserine sulfhydrylase/ *O*-succinylhomoserine sulfhydrylase	3.1	2.9	2.9	3	EC: 2.5.1.49/MetY	PF01053	Cysteine and methionine metabolism
IGTS8_peg770	Homoserine *O*-acetyltransferase	D	1.4	1.4	1.3	EC: 2.3.1.46, 2.3.1.31/MetXA	PF00561	Cysteine and methionine metabolism
IGTS8_peg771	*O*-Acetylhomoserine sulfhydrylase	D	D	D	D	EC: 2.5.1.49/MetY	PF01053	Cysteine and methionine metabolism
IGTS8_peg5771	*O*-Acetylhomoserine sulfhydrylase (EC 2.5.1.49/*O*-succinylhomoserine sulfhydrylase (EC 2.5.1.48)	D	D	-0.7	-0.8	MetZ	PF01053	Cysteine and methionine metabolism
IGTS8-peg5353	Cysteine synthase	0.8	0.3	D	0.4	EC: 2.5.1.47/CysK1	PF00291	Cysteine biosynthesis
IGTS8-peg5354	Serine acetyltransferase	D	D	D	D	EC: 2.3.1.30/CysE	PF00132	Cysteine biosynthesis
IGTS8-peg6007	Cysteine synthase B	1.0	D	0.9	2.1	EC: 2.5.1.47/CysK	PF00291	Cysteine biosynthesis
IGTS8-peg3011	Cystathionine gamma-lyase	D	-0.6	-0.6	-0.7	EC: 2.5.1.48/MetB	PF01053	Cysteine and methionine metabolism

Protein ID	Protein Annotation	Log2 Fold Change#				EC/TC No. /Protein Name	Pfam No.	Pathway/Proposed Function
		EL	ML	LL	SP			
IGTS8-peg3012	Cystathionine beta-synthase	D	D	-0.3	D	EC: 4.2.1.22/CBS	PF00291	Cysteine and methionine metabolism
IGTS8-peg1115	Cystathionine gamma-lyase	D	D	D	D	EC: 4.4.1.1/CTH	PF01053	Cysteine biosynthesis
IGTS8_peg5907	5-Methyltetrahydropteroyltriglutamate -homocysteine methyltransferase (cobalamin-independent)	8.1	7.6	7.6	7.7	EC: 2.1.1.14/MetE	PF01717	Methionine biosynthesis
IGTS8_peg1786	5-methyltetrahydrofolate-homocysteine methyltransferase	D	D	D	D	EC: 2.1.1.13/MetH	PF02965	Methionine biosynthesis
IGTS8_peg1600	S-adenosylmethionine synthetase	1.4	1.1	0.8	1.2	EC: 2.5.1.6/MetK	PF02773	Biosynthesis of S-adenosylmethionine
IGTS8_peg2690	Alpha-ketoglutarate-dependent taurine dioxygenase	D	2.2	D	1.3	EC: 1.14.11.17/TauD_7	PF02668	Sulfur acquisition
IGTS8_peg2683	Luciferase family protein	7.2	8	8.3	7.1	SsuD	PF00296	Sulfur acquisition
IGTS8_peg5938	Choline-sulfatase	D	DS	DS	DS	EC: 3.1.6.1/AtsA1	PF00884	Sulfur acquisition
IGTS8_peg5939	Alpha-ketoglutarate-dependent taurine dioxygenase	DS	DS	DS	D	EC: 1.14.11.17/TauD	PF02668	Sulfur acquisition
IGTS8_peg5940	Possible transcriptional regulator, ROK family	5.1	4.2	5	DS	--------------------	PF00480	Transcriptional regulator
IGTS8_peg4968	Ketopantoate reductase PanG	0.8	1.6	1.7	2.4	EC: 1.1.1.169/PanG	PF10727	CoA biosynthesis
IGTS8_peg4969	Pantoate-beta-alanine ligase	0.6	0.8	0.8	0.9	EC: 6.3.2.1/PanC	PF02569	CoA biosynthesis

Protein ID	Protein Annotation	Log$_2$ Fold Change[#]				EC/TC No. /Protein Name	Pfam No.	Pathway/Proposed Function
		EL	ML	LL	SP			
IGTS8_peg2998	Pantothenate kinase	2.2	1.6	1	1	EC: 2.7.1.33/CoaA	PF00845	CoA biosynthesis
IGTS8_peg1907	Dephospho-CoA kinase	0.9	1.7	1.5	1.8	EC: 2.7.1.24/CoaE	PF01121	CoA biosynthesis
IGTS8_peg1128	Phosphopantetheine adenylyltransferase	D	1	1.5	1.6	EC: 2.7.7.3/CoaD	PF01467	CoA biosynthesis
IGTS8_peg1599	Phosphopantothenoylcysteine decarboxylase /Phosphopantothenoylcysteine synthetase	0.8	0.7	0.7	0.8	EC:4.1.1.36/ 6.3.2.5/CoaBC	PF04127	CoA biosynthesis
IGTS8_peg5948	Glycosyltransferase MshA involved in mycothiol biosynthesis	1.2	0.9	1.4	DS	EC: 2.4.1.250/MshA	PF00534	Mycothiol biosynthesis
IGTS8-peg2930	*N*-acetyl-1-D-myo-inositýl-2-amino-2-deoxy-alpha- D-glucopyranoside deacetylase MshB	D	0.5	0.7	1.4	EC: 3.5.1.103/MshB	PF02585	Mycothiol biosynthesis
IGTS8_peg1793	L-Cysteine:1D-myo-inositol 2-amino-2-deoxy-alpha-D-glucopyranoside ligase MshC	D	0.6	0.9	1.1	EC: 3.3.1.13/MshC	PF01406	Mycothiol biosynthesis
IGTS8_peg3571	Acetyl-CoA: Cys-GlcN-Ins acetyltransferase, mycothiol synthase MshD	D	D	0.8	1.2	EC:2.3.1.189/MshD	PF00583	Mycothiol biosynthesis
IGTS8_peg4196	Putative hydrolase	D	-2.5	-3.3	-3.3	----------------	PF00753	Mycothiol metabolism
IGTS8_peg4197	Formaldehyde dehydrogenase MscR, NAD/mycothiol-dependent	D	D	0.5	0.7	EC:1.1.1.306/MscR	PF08240	Mycothiol metabolism

Protein ID	Protein Annotation	Log2 Fold Change[#]				EC/TC No. /Protein Name	Pfam No.	Pathway/Proposed Function
		EL	ML	LL	SP			
IGTS8_peg1232	NADPH-dependent mycothiol reductase Mtr	D	D	D	0.7	EC: 1.8.1.15/Mtr	PF07992	Mycothiol metabolism
IGTS8-peg3003	Mycothiol S-conjugate amidase Mca	D	0.7	1	1.4	EC: 3.5.1.115/Mca	PF02585	Mycothiol metabolism
IGTS8_peg4243	Coenzyme F420-dependent N5,N10-methylene tetrahydromethanopterin reductase and related flavin-dependent oxidoreductases; sulfonate monooxygenase	10.6	10.4	9.5	8.7	EC: 1.14.14.35/SfnG	PF00296	Sulfur acquisition
IGTS8_peg4062	Alkanesulfonate monooxygenase	4	3.6	3	3.2	EC: 1.14.14.5/SsuD	PF00296	Sulfur acquisition
IGTS8_peg3074	Dimethylhistidine N-methyltransferase	D	D	1.9	2.2	EC: 2.1.1.44/EgtD	PF10017	Ergothioneine biosynthesis
IGTS8_peg3075	Glutamine amidotransferases class-II	D	D	D	2.3	EC: 3.5.1.118/EgtC	PF13522	Ergothioneine biosynthesis
IGTS8_peg3076	Serine/threonine kinase	D	D	1.2	1.5	EC: 1.14.99.50/EgtB	PF03781	Ergothioneine biosynthesis
IGTS8_peg3077	Glutamate-cysteine ligase	D	1.2	2	1.7	EC: 6.3.2.2/EgtA	PF04107	Ergothioneine biosynthesis
IGTS8_peg3078	Putative amino acid transporter	ND	ND	ND	ND	TC: 2.A.3.2/PlaP	PF13520	Amino acid permease

Protein ID	Protein Annotation	Log2 Fold Change#				EC/TC No. /Protein Name	Pfam No.	Pathway/Proposed Function
		EL	ML	LL	SP			
IGTS8_peg3079	Cysteine desulfurase	D	D	1.2	1.7	EC: 2.8.1.7/IscS	PF00266	Thiamin and molybdenum cofactor biosynthesis, sulfur relay system
IGTS8_peg3146	Molybdopterin biosynthesis protein MoeA	D	1.1	0.8	0.9	EC: 2.10.1.1/MoeA	PF00994	Molybdenum cofactor biosynthesis
IGTS8_peg3153	Molybdenum cofactor biosynthesis protein	D	2.1	2.7	2.9	MoaB	PF00994	Molybdenum cofactor biosynthesis
IGTS8_peg1034	Molybdenum cofactor biosynthesis protein MoaC/Molybdenum cofactor biosynthesis protein MoaB	0.8	D	0.8	1.4	EC: 4.6.1.17/MoaCB	PF00994	Molybdenum cofactor biosynthesis
IGTS8_peg1035	Molybdopterin biosynthesis protein MoeA	D	1.3	1.2	1.6	EC: 2.10.1.1/MoeA	PF03453	Molybdenum cofactor biosynthesis
IGTS8_peg5782	Amino acid ABC transporter permease protein	D	D	D	D	TC: 3.A.1.3/TcyB	PF00528	Probable cystine transporter
IGTS8_peg5783	Methionine ABC transporter ATP-binding protein	DS	DS	DS	DS	EC: 7.4.2.1/TcyC	PF00528	Probable cystine transporter
IGTS8_peg5784	Periplasmic binding protein	D	DS	5.8	3.6	TC: 3.A.1.3/FliY/TcyA	PF00497	Probable cystine transporter
IGTS8_peg5785	Sarcosine oxidase	DS	D	DS	DS	EC: 1.5.3.1/1.5.3.7/SoxA_1	PF01266	Glycine, Serine, threonine metabolism

Protein ID	Protein Annotation	Log$_2$ Fold Change#				EC/TC No. /Protein Name	Pfam No.	Pathway/Proposed Function
		EL	ML	LL	SP			
IGTS8_peg394	Predicted transcriptional regulator of sulfate adenylyltransferase	DS	DS	DS	DS	CymR/Rrf2 family member	PF02082	Probable transcriptional regulator for cysteine metabolism
IGTS8_peg1918	Thiosulfate sulfurtransferase, rhodanese	-2.1	-2.5	-1.9	-2.8	TrxA	PF00085	Thioredoxin
IGTS8_peg999	Potassium efflux system KefA protein/Small-conductance mechanosensitive channel	D	2.6	2.2	D	TC: 1.A.23.3/KefA	PF00924	Probable 2-HBP efflux
IGTS8_peg2369	Magnesium and cobalt efflux protein CorC	D	D	0.8	DS	CorC	PF03471	Probable 2-HBP efflux
IGTS8_peg751	Possible rhodanese-related sulfurtransferase	-2	-2.4	-1.7	-1.9	EC: 2.8.1.1/PspE	PF00581	Sulfurtransferase
IGTS8_peg3535	Nitrilotriacetate monooxygenase component A	8.5	7.4	6.8	5.8	EC: 1.14.13.-	PF00296	Sulfur acquisition
IGTS8_peg5732	Nitrilotriacetate monooxygenase component A	DS	D	DS	D	EC: 1.14.13.-/ScmK	PF00296	Sulfur acquisition
IGTS8_peg1647	ABC-type multidrug transport system, ATPase component	2.4	3.8	3.7	D	TC: 3.A.1/DrrA_5	PF00005	Probable 2-HBP efflux
IGTS8_peg5812	Thiazole biosynthesis protein ThiG	D	1.5	1.4	1.8	EC: 2.8.1.10/ThiG	PF05690	Thiamin/thiazole metabolism
IGTS8_peg2270	Hydroxyethylthiazole kinase	0.7	1.7	1.7	1.4	EC: 2.7.1.50/ThiM	PF02110	Thiamin/thiazole metabolism

Protein ID	Protein Annotation	Log2 Fold Change[#]				EC/TC No. /Protein Name	Pfam No.	Pathway/Proposed Function
		EL	ML	LL	SP			
IGTS8_peg951	Sulfur carrier protein adenylyltransferase	0.7	0.7	0.6	0.7	ThiF	PF00899	Thiamin/thiazole metabolism
IGTS8_peg1463	1-Deoxy-D-xylulose 5-phosphate synthase	0.9	1	1	1.1	EC: 2.2.1.7/Dxs	PF13292	Thiamin/thiazole metabolism
IGTS8_peg5816	Hydroxymethylpyrimidine phosphate kinase	D	D	0.7	1.6	EC: 2.7.4.7/ThiD	PF08543	Thiamin/thiazole metabolism
IGTS8_peg2644	Thiaminase II involved in salvage of thiamine pyrimidine moiety	-1.5	D	D	D	EC: 3.5.99.2/TenA	PF03070	Thiamin/thiazole metabolism
IGTS8_peg1119	Thiamin monophosphate kinase	D	D	D	D	EC: 2.7.4.16/ThiL	PF00586	Thiamin/thiazole metabolism
IGTS8_peg4214	3-Oxoacyl-[acyl-carrier protein] reductase	0.5	0.8	1.3	1.2	EC: 1.1.1.100/FabG	PF13561	Biotin biosynthesis
IGTS8_peg4825	Adenosylmethionine-8-amino-7-oxononanoate aminotransferase	D	D	D	D	EC: 2.6.1.62/BioA	PF00202	Biotin biosynthesis
IGTS8_peg2082	Dethiobiotin synthetase	ND	ND	ND	ND	EC: 6.3.3.3/BioD	PF13500	Biotin biosynthesis
IGTS8_peg2083	8-Amino-7-oxononanoate synthase	ND	ND	ND	ND	EC: 2.3.1.47/BioF	PF00155	Biotin biosynthesis
IGTS8_peg2084	Adenosylmethionine-8-amino-7-oxononanoate aminotransferase	ND	ND	ND	ND	EC: 2.6.1.62/BioA	PF00202	Biotin biosynthesis
IGTS8_peg2066	Biotin synthase	ND	ND	ND	ND	EC: 2.8.1.6/BioB	PF06968	Biotin biosynthesis
pIGTS8_peg6211	3-Oxoacyl-[acyl-carrier-protein]synthase KASII	D	D	-0.4	-0.4	EC: 2.3.1.179/FabF	PF00109	Biotin biosynthesis

Protein ID	Protein Annotation	Log2 Fold Change#				EC/TC No. /Protein Name	Pfam No.	Pathway/Proposed Function
		EL	ML	LL	SP			
IGTS8_peg880	Biotin-protein ligase	1	1.4	1.3	1.5	EC: 6.3.4.15/BirA	PF03099	BirA family transcriptional regulator, biotin operon repressor
IGTS8_peg2518	[Acyl-carrier-protein] acetyl transferase of FASI (EC 2.3.1.38) / Enoyl-[acyl-carrier-protein] reductase of FASI (EC 1.3.1.9) / 3-hydroxypalmitoyl-[acyl-carrier-protein] dehydratase of FASI (EC 4.2.1.61) / [Acyl-carrier-protein] malonyl transferase of FASI (EC 2.3.1.39) / [Acyl-carrier-protein] palmitoyl transferase of FASI (EC 2.3.1.-) / Acyl carrier protein of FASI / 3-oxoacyl-[acyl-carrier-protein] reductase of FASI (EC 1.1.1.100) / 3-oxoacyl-[acyl-carrier-protein] synthase of FASI (EC 2.3.1.41)	1.3	1.3	1	1.9	KasA_1	PF08354 PF00698 PF00109 PF02801 PF0157 PF18094 PF16073	Fatty acid and biotin biosynthesis
IGTS8_peg1417	Alkylhydroperoxide reductase protein C	1.4	2.6	2.6	2.4	EC: 1.11.1.28/AhpC	PF00578	Oxidative stress response
IGTS8_peg1416	Alkylhydroperoxidase protein D	D	D	D	D	EC: 1.11.1.28/AhpD	PF02627	Oxidative stress response
IGTS8_peg1040	5'-Methylthioadenosine nucleosidase (EC 3.2.2.16)/S-adenosylhomocysteine nucleosidase (EC 3.2.2.9)	D	D	D	D	MtnN	PF01048	SAM degradation, methionine salvage

Protein ID	Protein Annotation	Log2 Fold Change[#]				EC/TC No. /Protein Name	Pfam No.	Pathway/Proposed Function
		EL	ML	LL	SP			
IGTS8_peg2566	Homoserine kinase	D	D	1	0.8	EC: 2.7.1.39/ThrB	PF00742	Methionine biosynthesis
IGTS8_peg2568	Homoserine dehydrogenase	D	D	D	D	EC: 1.1.1.3/Hom	PF0288	Methionine biosynthesis
IGTS8_peg4995	2-Hydroxychromene-2-carboxylate isomerase/DsbA-like thioredoxin domain	-2.3	-2.3	-1.2	-1.2	DsbA	PF01323	Protein-disulfide isomerase
IGTS8_peg2514	Thiol peroxidase, Bcp-type	D	0.8	1.2	1.8	EC:1.11.124	PF00578	Oxidative stress response
IGTS8_peg5768	Ferredoxin-NADP(+) reductase, actinobacterial (eukaryote-like) type	D	1	1	1	EC: 1.18.1.2/FprA_1	PF07992	Inorganic sulfur assimilation
IGTS8_peg3374	Phosphoserine aminotransferase	D	D	D	D	EC: 2.6.1.52/SerC	PF00266	Phosphoserine biosynthesis
IGTS8_peg352	D-3-Phosphoglycerate dehydrogenase	-1.8	D	-1.9	D	EC: 1.1.1.29	PF02826	Phosphoserine biosynthesis
IGTS8_peg1100	D-3-Phosphoglycerate dehydrogenase	0.7	0.9	0.9	1	EC: 1.1.1.95/SerA	PF02826	Phosphoserine biosynthesis
IGTS8_peg5974	Uroporphyrinogen-III methyltransferase/ Uroporphyrinogen-III synthase	0.8	0.6	0.6	0.8	EC: 2.1.1.107, 4.2.1.75/CysG	PF02602	Siroheme biosynthesis
IGTS8_peg1241	Siroheme synthase / Precorrin-2 oxidase/ Sirohydrochlorin ferrochelatase/ Uroporphyrinogen-III methyltransferase	D	D	D	D	EC: 2.1.1.107, 4.99.1.4/CysG	PF00590	Siroheme biosynthesis

Protein ID	Protein Annotation	Log2 Fold Change#				EC/TC No. /Protein Name	Pfam No.	Pathway/Proposed Function
		EL	ML	LL	SP			
IGTS8_peg2581	Probable CysQ protein	D	ND	D	D	EC:3.1.3.7/CysQ	PF00459	Dephosphorylation of 3'-phosphoadenosine 5'-phosphate
IGTS8_peg2618	Hypothetical protein	ND	ND	ND	ND	-------------------	PF00174	Probable sulfite oxidase
IGTS8_peg446	Putative membrane protein YeiH	ND	ND	ND	ND	--------------------	PF03601	Sulfate efflux

*Log_2 fold change values are given for proteins showing *p*-values lower than 0.05 and identified with at least 5 unique peptides. D: protein was identified but not confidently quantified in the condition, ND: protein was not detected in the condition, DS: protein was uniquely identified in dibenzothiophene cultures but not detected in the sulfate-culture. 2-HBP: 2-hydroxybiphenyl, SAM: S-adenosylmethionine, SSI: Sulfate Starvation-Induced.
Growth phase: EL: early-log, ML: mid-log, LL: late-log, SP: stationary phase

Table S4: Cysteine and methionine content (%) in some differentially produced proteins*

Protein ID	Protein Name	Protein Length (aa)#	%Cysteine	%Methionine
IGTS8_peg167	Probable sulfatase	456	0.4	0.66
IGTS8_peg168	Sulfate adenylyltransferase subunit 1 (EC 2.7.7.4)/ Adenylylsulfate kinase (EC 2.7.1.25) (CysNC)	626	0.3	0.3
IGTS8_peg169	Sulfate adenylyltransferase subunit 2	304	0.0	0.66
IGTS8_peg170	Sulfotransferase (Stf0)	250	0.0	0.8
IGTS8_peg352	D-3-phosphoglycerate dehydrogenase (EC 1.1.1.95)	308	0.3	1.0
IGTS8_peg751	Possible rhodanese-related sulfurtransferase (PspE)	102	1.0	1.0
IGTS8_peg1115	Cystathionine gamma-lyase (EC 4.4.1.1) (CTH)	383	0.8	1.8
IGTS8_peg1786	5-Methyltetrahydrofolate-homocysteine methyltransferase (MetH)	1163	0.9	2.4
IGTS8_peg1918	Thiosulfate sulfurtransferase, rhodanese (EC 2.8.1.1)) (TrxA)	123	1.6	1.6
IGTS8_peg2383	Ferredoxin--sulfite reductase, actinobacterial type (EC 1.8.7.1) (Sir)	576	1.2	0.9
IGTS8_peg2384	Phosphoadenylylsulfate reductase [thioredoxin]/Adenylyl-sulfate reductase [thioredoxin] (CysH)	237	2.1	0.8
IGTS8_peg2385	Sulfate adenylyltransferase subunit 2 (EC 2.7.7.4) (CysD)	307	0.0	1.0
IGTS8_peg2386	Sulfate adenylyltransferase subunit 1 (EC 2.7.7.4) (CysN)	428	0.2	0.0
IGTS8_peg2387	Sirohydrochlorin cobaltochelatase (EC 4.99.1.3) (CbiX-SirB)	251	0.8	0.8
IGTS8_peg3011	Cystathionine gamma-lyase (EC 4.4.1.1) (MetB)	390	0.8	1.8
IGTS8_peg3012	Cystathionine beta-synthase (EC 4.2.1.22) (CBS)	461	0.9	1.7
IGTS8_peg3535	Nitrilotriacetate monooxygenase component A	464	0.0	0.0
IGTS8_peg3888	*O*-acetylhomoserine sulfhydrylase/ *O*-succinylhomoserine sulfhydrylase (MetY)	437	0.0	0.2
IGTS8_peg4196	Putative hydrolase in cluster with formaldehyde/S-nitrosomycothiol reductase MscR	208	1.0	0.5
IGTS8_peg4243	Coenzyme F420-dependent N5,N10-methylene tetrahydromethanopterin reductase and related flavin-dependent oxidoreductases; sulfonate monooxygenase SfnG	378	0.0	0.5
IGTS8_peg4995	2-Hydroxychromene-2-carboxylate isomerase/DsbA-like thioredoxin domain	209	1	1.4
IGTS8_peg5353	Cysteine synthase (EC 2.5.1.47)(CysK1)	311	0.3	1.6
IGTS8_peg5732	Nitrilotriacetate monooxygenase component A	450	0.0	0.2
IGTS8_peg5771	*O*-Acetylhomoserine sulfhydrylase (EC 2.5.1.49)/O-succinylhomoserine sulfhydrylase (MetZ)	407	0.7	2.0
IGTS8_peg5907	5-Methyltetrahydropteroyltriglutamate -homocysteine methyltransferase (cobalamin-independent) (MetE)	768	0.3	0.9
IGTS8_peg5938	Choline-sulfatase	773	0.1	0.9
IGTS8_peg5939	Alpha-ketoglutarate-dependent taurine dioxygenase	325	0.0	0.0
IGTS8_peg6007	Cysteine synthase (CysK)	367	1.1	1.4
pIGTS8_peg6351	Dibenzothiophene desulfurization enzyme A (DszA)	447	0.0	1.1
pIGTS8_peg6352	Dibenzothiophene desulfurization enzyme B (DszB)	365	0.3	0.0
pIGTS8_peg6353	Acyl-CoA dehydrogenase; probable dibenzothiophene desulfurization enzyme (DszC)	417	0.2	0.7

*See Table S1 for protein expression profiles, #Amino acids
